# Genetic and Pharmacological Modifications of Thrombin Formation in Apolipoprotein E-deficient Mice Determine Atherosclerosis Severity and Atherothrombosis Onset in a Neutrophil-Dependent Manner

**DOI:** 10.1371/journal.pone.0055784

**Published:** 2013-02-07

**Authors:** Julian I. Borissoff, Jeroen J. T. Otten, Sylvia Heeneman, Peter Leenders, René van Oerle, Oliver Soehnlein, Sarah T. B. G. Loubele, Karly Hamulyák, Tilman M. Hackeng, Mat J. A. P. Daemen, Jay L. Degen, Hartmut Weiler, Charles T. Esmon, Joanne van Ryn, Erik A. L. Biessen, Henri M. H. Spronk, Hugo ten Cate

**Affiliations:** 1 Laboratory for Clinical Thrombosis and Hemostasis, Department of Internal Medicine, Cardiovascular Research Institute Maastricht, Maastricht University Medical Center, Maastricht, The Netherlands; 2 Program in Cellular and Molecular Medicine, Boston Children's Hospital, Harvard Medical School, Boston, Massachusetts, United States of America; 3 Department of Pediatrics, Harvard Medical School, Boston, Massachusetts, United States of America; 4 Department of Pathology, Experimental Vascular Pathology Research Group, Cardiovascular Research Institute Maastricht, Maastricht University, Maastricht, The Netherlands; 5 Institute for Cardiovascular Molecular Research, Medical Faculty, RWTH Aachen University, Aachen, Germany; 6 Department of Biochemistry, Cardiovascular Research Institute Maastricht, Maastricht University Medical Center, Maastricht, The Netherlands; 7 Developmental Biology, Children’s Hospital Research Foundation, University of Cincinnati College of Medicine, Cincinnati, Ohio, United States of America; 8 Blood Research Institute, The Blood Center of Southeastern Wisconsin, Milwaukee, United States of America; 9 Oklahoma Medical Research Foundation and Howard Hughes Medical Institute, Oklahoma City, Oklahoma, United States of America; 10 Department of CardioMetabolic Disease Research, Boehringer Ingelheim Pharma GmbH & Co. KG, Biberach an der Riss, Germany; Leiden University Medical Center, The Netherlands

## Abstract

**Background:**

Variations in the blood coagulation activity, determined genetically or by medication, may alter atherosclerotic plaque progression, by influencing pleiotropic effects of coagulation proteases. Published experimental studies have yielded contradictory findings on the role of hypercoagulability in atherogenesis. We therefore sought to address this matter by extensively investigating the *in vivo* significance of genetic alterations and pharmacologic inhibition of thrombin formation for the onset and progression of atherosclerosis, and plaque phenotype determination.

**Methodology/Principal Findings:**

We generated transgenic atherosclerosis-prone mice with diminished coagulant or hypercoagulable phenotype and employed two distinct models of atherosclerosis. Gene-targeted 50% reduction in prothrombin (FII^−/WT^:ApoE^−/−^) was remarkably effective in limiting disease compared to control ApoE^−/−^ mice, associated with significant qualitative benefits, including diminished leukocyte infiltration, altered collagen and vascular smooth muscle cell content. Genetically-imposed hypercoagulability in TM^Pro/Pro^:ApoE^−/−^ mice resulted in severe atherosclerosis, plaque vulnerability and spontaneous atherothrombosis. Hypercoagulability was associated with a pronounced neutrophilia, neutrophil hyper-reactivity, markedly increased oxidative stress, neutrophil intraplaque infiltration and apoptosis. Administration of either the synthetic specific thrombin inhibitor Dabigatran etexilate, or recombinant activated protein C (APC), counteracted the pro-inflammatory and pro-atherogenic phenotype of pro-thrombotic TM^Pro/Pro^:ApoE^−/−^ mice.

**Conclusions/Significance:**

We provide new evidence highlighting the importance of neutrophils in the coagulation-inflammation interplay during atherogenesis. Our findings reveal that thrombin-mediated proteolysis is an unexpectedly powerful determinant of atherosclerosis in multiple distinct settings. These studies suggest that selective anticoagulants employed to prevent thrombotic events may also be remarkably effective in clinically impeding the onset and progression of cardiovascular disease.

## Introduction

Blood coagulation and inflammation are evolutionary coupled host-defense mechanisms, which operate via common molecular and cellular pathways, serve as protection against infections or bleeding, promote wound healing and restore the integrity of injured tissues [1]–[3]. Atherosclerosis is a progressive chronic inflammatory vascular disorder, which can result in atherosclerotic plaque rupture and subsequent superimposed thrombus formation [4]–[6]. Besides the detrimental role of coagulation during the onset of acute atherothrombotic complications, there is evidence that local activation of hemostatic factors within early human atherosclerotic lesions may also be important in atherogenesis [7]. In addition to the overt leukocyte infiltration into the lesions and enhanced cell death, which are considered major markers for plaque instability, today’s concept of a vulnerable plaque suggests that repeated plaque microruptures and subclinical microthrombosis are critical processes to plaque growth and subsequent atherothrombosis [8]–[10]. Histopathological reports demonstrate that thrombi may exist prior to rupture [11], [12]. Numerous *in vitro* studies indicate that key clotting proteases such as thrombin can also catalyze a wide range of cellular actions related to cardiovascular function and pathophysiology - e.g. vascular permeability, oxidative stress, migration and proliferation of vascular smooth muscle cells, leukocyte adhesion, chemotaxis, inflammation, and apoptosis [13]. Experimental animal studies demonstrate that administration of direct thrombin inhibitors in ApoE^−/−^ mice attenuates atherosclerotic plaque progression and promotes plaque stability of advanced atherosclerotic lesions by reducing the levels of inflammation and the number of macrophages infiltrating the lesions [14]–[16]. In sharp contrast, there is also evidence showing that hypercoagulability in ApoE^−/−^ mice carrying prothrombotic mutations promotes atherosclerotic plaque stability via thrombin-mediated impairment of monocyte transendothelial migration [17]. In the near future, millions of patients with arterial vascular disease will be treated with novel, selective anticoagulant agents. Whereas this matter remains of major scientific and clinical significance, there is still limited understanding of the relevance of blood coagulation in atherosclerosis *in vivo* [18]. In attempting to reconcile these apparently contradictory findings, we extensively investigated the *in vivo* significance of genetic alterations and pharmacologic inhibition of thrombin formation for the onset and progression of atherosclerosis, but also plaque phenotype determination.

## Methods

### Animals

TM^Pro/Pro^ mice, carrying a thrombomodulin (TM) gene mutation resulting in diminished TM-dependent generation of activated protein C (APC) [19], and prothrombin (FII) heterozygous mice with genetically imposed hypoprothrombinemia [20] were crossed into a pure C57BL/6 background for at least 8 generations and subsequently crossbred to ApoE^−/−^ mice (Charles River, Maastricht, The Netherlands), carrying the same background. Only female mice were used throughout the entire study. All animal experimental protocols were carried out in compliance with the Dutch government guidelines and were approved by the Animal Care and Use Committee of Maastricht University (Maastricht, The Netherlands).

### Mouse Models of Atherosclerosis

In a spontaneous atherosclerosis model, female TM^Pro/Pro^:ApoE^−/−^, FII^−/WT^:ApoE^−/−^ (age, 8–9 weeks; n = 10 per group) and control ApoE^−/−^ mice (age, 8–9 weeks; n = 20) received regular chow diet (Hope Farms, Woerden, The Netherlands) for 35 weeks and were then sacrificed for a detailed analysis. In a separate experimental setup, consisting of identical groups, carotid atherosclerotic plaques were induced via placement of perivascular collars around the common carotid arteries as described before [21]. All animals were fed on a high-fat diet (15% cocoa butter, 1% corn oil, 0.25% cholesterol, 40.5% sucrose, 10% cornstarch, 20% casein, free of cholate, total fat content 16%; Hope Farms, Woerden, The Netherlands) for two weeks before collar placement and for additional six weeks after surgery. Diets and water were provided *ad libitum* throughout all experiments.

### Pharmacological Interventions

Female TM^Pro/Pro^:ApoE^−/−^ mice (n = 10 per treatment group; age, 8–9 weeks) fed on a standard high-fat diet (D12451; Research Diets, NJ, USA) for 2 weeks were subsequently subjected to a surgical implantation of non-constrictive perivascular carotid collars and then assigned to different interventions or placebo for a total of 6 weeks. The study design involved an intervention arm with mice receiving standard D12451 high-fat chow supplemented with oral Dabigatran etexilate (7.5 mg DE/gram chow). In a second intervention arm, TM^Pro/Pro^:ApoE^−/−^ mice were fed on a standard D12451 high-fat diet and received intraperitoneal (i.p.) administration of recombinant murine APC (rmAPC)) in bolus doses of 2.5 mg/kg/per every 5 days. Placebo-treated mice received injection of saline and were fed on standard D12451 high-fat chow. rmAPC was produced in the laboratory of Dr. Charles T. Esmon (Oklahoma Medical Research Foundation and Howard Hughes Medical Institute, Oklahoma City, Oklahoma, USA). Both DE-supplemented high-fat and placebo chow diets were prepared at Department of CardioMetabolic Disease Research, Boehringer Ingelheim Pharma GmbH & Co. KG (Biberach an der Riss, Germany).

### Blood Sampling, Blood Cell Counts, Blood Coagulation and Lipid Profile Analysis. Cytokines and Chemokines Profile Analysis. Tissue Harvesting, Preparation and Morphometry. Histology and Immunohistochemistry. FeCl_3_-induced Carotid Artery Injury Model. Lipid Uptake Analysis in Bone Marrow-derived Macrophages (BMM). Leukocyte-Endothelium Interactions in Atherosclerotic Carotid Arteries. Characterization of Bone Marrow Cell Populations by CFU-C Assays and Flow Cytometry

For an expanded Methods section, please see the online supplement of the article (Methods S1).

### Statistical Analysis

All statistics were performed using Prism, version 6.00 (GraphPad Software Inc., San Diego, CA, USA) and IBM SPSS Statistics 20.0 (SPSS Japan Inc., an IBM company, Tokyo, Japan). Data sets were assessed for normality using Kolmogorov-Smirnov test or Bartlett’s test for homogeneity of variance. Data were compared using unpaired 2-tailed *t* test or one-way ANOVA, followed by Newman-Keuls posthoc test for multiple comparisons. In case of non-normal distribution, non-parametric tests such as Mann-Whitney or Kruskall-Wallis test with Dunn’s post hoc analysis were used as appropriate. Data are expressed as mean ± SD, unless otherwise stated. A 2-tailed p<0.05 was considered statistically significant.

## Results

We first generated transgenic cross breds with genetically imposed variations in coagulation potential. Homozygous prothrombin (FII) deficiency in mice results in embryonic and neonatal lethality due to severe hemorrhagic phenotype and loss of vascular integrity [20]. Therefore, we employed uniformly viable FII heterozygous ApoE^−/−^ mice (characterized by hypoprothrombinemia and diminished FVII and thrombin generation but no spontaneous bleeding risk; *Table S1A*) in comparative studies with control, prothrombin-sufficient ApoE^−/−^ mice. TM^Pro/Pro^ mice carry a point mutation in the thrombomodulin (TM) gene, which impairs TM-dependent generation of the natural anticoagulant activated protein C (APC) [19]. TM^Pro/Pro^:ApoE^−/−^ mice demonstrated a profound hypercoagulable state with substantially increased plasma thrombin generation and fibrinogen levels, but also significantly higher factor FVII, FX, and FVIII levels (*Table S1A*).

### Coagulation Phenotype is a Key Factor in Atherosclerotic Plaque Growth and Phenotype Determination

**Figure 1:**
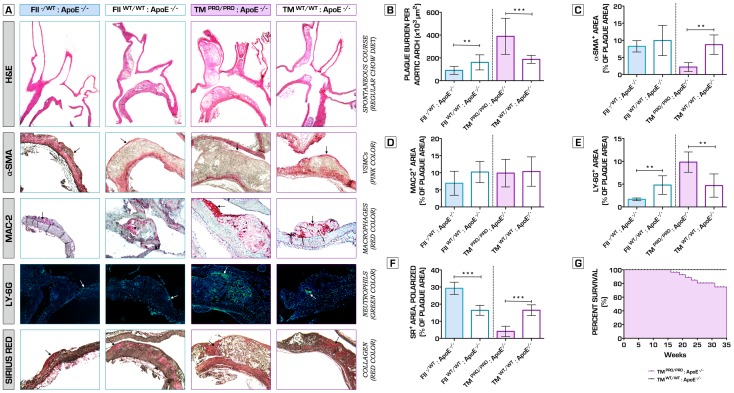
The effects of variations in coagulation potential on atherogenesis in a spontaneous atherosclerosis model at 35 weeks on a regular chow diet. (A) Top row represents images of the aortic arch and its main branches, stained with hematoxylin and eosin (H&E), used to analyze the extent of atherosclerotic plaque burden. To determine plaque phenotype characteristics, sections were stained against α-smooth muscle actin (vascular smooth muscle cell content – second row), MAC-2^+^ (macrophage infiltration – third row), Ly-6G (neutrophil recruitment – fourth row) and with Sirius red (collagen – bottom row). (B) Hypocoagulability in FII^−/+^:ApoE^−/−^ significantly attenuated atherosclerosis plaque development (90.6±35.1*10^3^ µm^2^ total plaque burden) when compared to normal ApoE^−/−^ mice (160.6±65.9*10^3^ µm^2^)(n = 10 per group, p = 0.0084). Total plaque area in TM^Pro/Pro^:ApoE^−/−^ mice was established 389.1±158.4*10^3^ vs. 187.0±35.1*10^3^ µm^2^ in the corresponding control ApoE^−/−^ group (n = 10 per group, p = 0.0010). (C) TM^Pro/Pro^:ApoE^−/−^ mice atherosclerotic plaques demonstrated a significant decrease in intimal vascular smooth muscle cell content (2.2±1.3% of plaque area) compared to ApoE^−/−^ mice (8.7±2.9% of plaque area)(n = 10 per group, p = 0.0016). Recruitment of macrophages within the lesions did not differ between all experimental groups (D). Neutrophil infiltration was significantly diminished in the lesions of hypocoagulable FII^−/+^:ApoE^−/−^ mice (n = 10 per group, p = 0.0092 vs. ApoE^−/−^ mice), and substantially increased in the TM^Pro/Pro^:ApoE^−/−^ intima (n = 10 per group, p = 0.0094 vs. ApoE^−/−^ mice) (E). A similar trend was observed with regard to collagen deposition within the atherosclerotic plaques. In FII^−/+^:ApoE^−/−^ mice, 29.3±3.6% of the plaque area stained collagen-positive (n = 10 per group, p = 0.0002 vs. ApoE^−/−^ mice). In contrast, Sirius red staining showed only 4.1±3.0% positivity for collagen in the TM^Pro/Pro^:ApoE^−/−^ lesions (n = 10 per group, p = 0.0002 vs. ApoE^−/−^ mice) (F). By 35 weeks (established duration of the experiment), we recorded the following fatal events: 6 of 16 TM^Pro/Pro^:ApoE^−/−^, 1 of 11 FII^−/+^:ApoE^−/−^ and 0 of 20 ApoE^−/−^ control mice. Dead mice were not included from the study analyses. The exact cause of death remained unclear. Kaplan-Meier analysis of the survival data comparing TM^Pro/Pro^:ApoE^−/−^ vs. ApoE^−/−^ mice, as determined by the Gehan-Breslow-Wilcoxon test, indicated that hypercoagulability is linked to significantly higher spontaneous mortality rates (p = 0.0165) (G). No significant difference was found between FII^−/+^:ApoE^−/−^ and ApoE^−/−^ control mice (p = 0.3173) (data not shown).*p<0.05; **p<0.01; ***p<0.001. Error bars represent mean ± SD. Arrows indicate examples of positive staining. Abbreviations: H&E – hematoxylin and eosin; α-SMA - α-smooth muscle actin; SR – Sirius red.

We then assessed the extent, as well as the phenotype of the atherosclerotic plaques formed in the aortic arch in experimental cohorts of mice following 35 weeks on a regular chow diet. FII^−/+^:ApoE^−/−^ mice with a genetic deficit in prothrombin exhibited highly attenuated atherosclerotic lesion formation relative to control ApoE^−/−^ mice (*Figure 1A,B*). Macrophage infiltration (MAC-2^+^ cells) and α-smooth muscle actin (SMA; α-SMA^+^ cells) content were unaffected in FII^−/+^:ApoE^−/−^ mice lesions compared to ApoE^−/−^ control mice (*Figure 1A,C,D*). However, hypoprothrombinemia was also linked to a significant decrease in neutrophil recruitment (*Figure 1A,E*), abundant collagen deposition (*Figure 1A,F*), thus showing a more fibrotic appearance, stable plaque phenotype and decreased number of advanced atherosclerotic lesions formed. In sharp contrast, pro-thrombotic TM^Pro/Pro^:ApoE^−/−^ mice displayed severe atherosclerosis development with remarkably increased total plaque area (*Figure 1A,B*). TM^Pro/Pro^:ApoE^−/−^ mice showed unstable lesions (*Figure 1A,B*), associated with markedly decreased α-SMA and collagen content (*Figure 1A,C,F*), and significantly higher neutrophil (Ly6G^+^ cells) infiltration (*Figure 1A,E*). These effects were independent of plasma lipid levels (*Table S1B*) and could not be attributed to an increased uptake of modified lipoproteins by macrophages (*Figure S1A,B,C,D*). Hypercoagulable TM^Pro/Pro^:ApoE^−/−^ mice showed significantly increased spontaneous mortality rates, albeit that the exact cause of death could not be pinpointed (*Figure 1G*).

**Figure 2:**
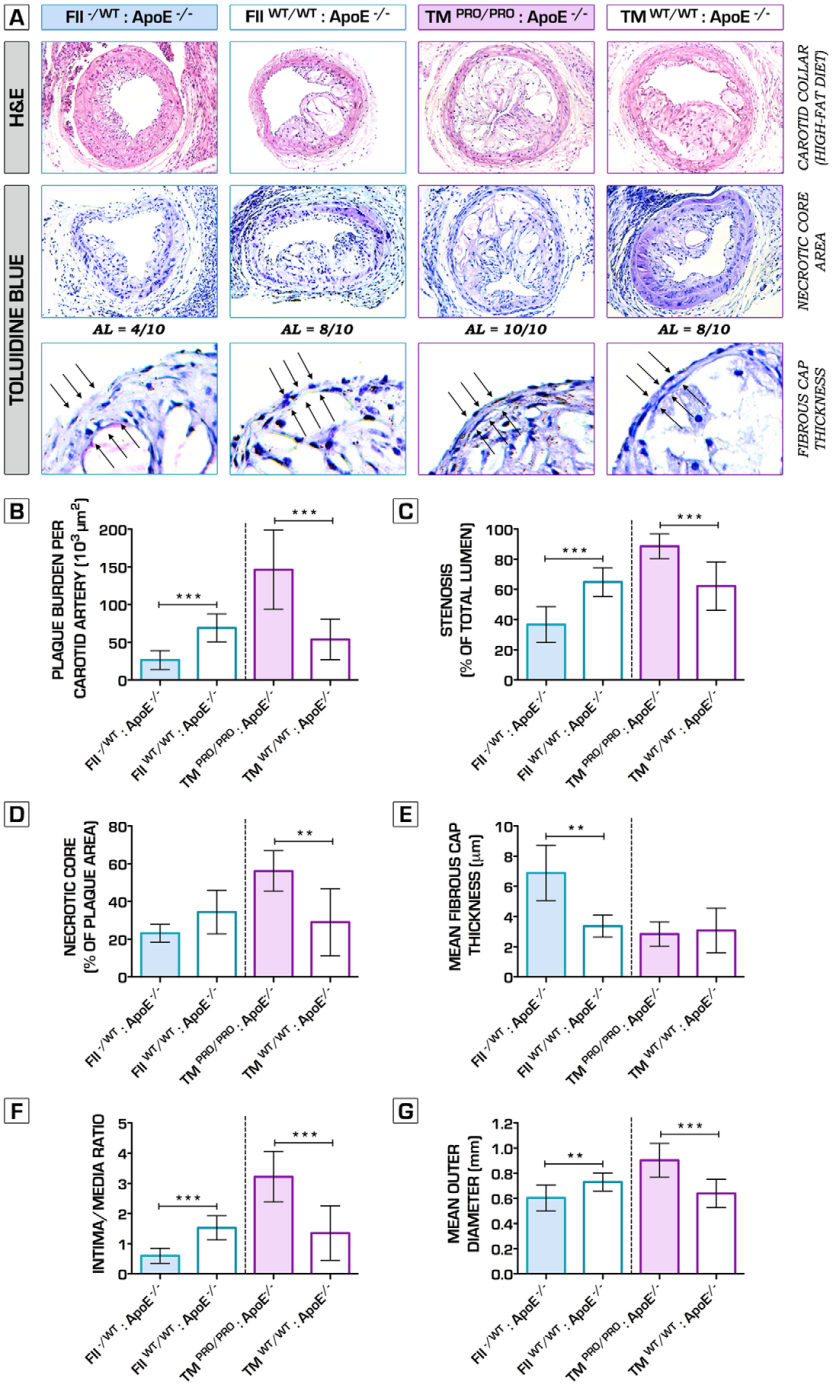
Morphometrical analysis of periadventitial cuff-induced atherosclerosis in mice with genetically imposed alterations in blood coagulation potential. (A) Representative hematoxylin and eosin (H&E)-stained sections of carotid arteries of FII^−/+^:ApoE^−/−^, TM^Pro/Pro^:ApoE^−/−^ and control ApoE^−/−^ mice (top row). Necrotic core areas of the atherosclerotic lesions were identified and quantified by using toluidine blue (TB) staining (second and third row). (B, C) Whereas hypocoagulable mice were significantly protected against plaque progression (26.5±12.6*10^3^ in FII^−/+^:ApoE^−/−^ vs. 69.2±18.4*10^3^ µm^2^ in ApoE^−/−^ control mice, n = 10 per group, p<0.0001), pro-thrombotic mice developed severe and occlusive atherosclerotic burden (146.4±52.7*10^3^ in TM^Pro/Pro^:ApoE^−/−^ vs. 53.9±27.0*10^3^ µm^2^ in ApoE^−/−^ control mice, n = 10 per group, p = 0.0001). The degree of stenosis in TM^Pro/Pro^:ApoE^−/−^ reached an average of 88.6±8.1% (vs. 62.2±16.1% in ApoE^−/−^ mice, n = 10 per group, p = 0.0002), whereas it was substantially lower in FII^−/+^:ApoE^−/−^ mice (36.8±11.9% vs. 64.9±9.6% in ApoE^−/−^ mice, n = 10 per group, p<0.0001). (A, D) Pearson's chi-squared test (*χ* ^2^) detected a significant difference in the number of advanced atherosclerotic lesions (presence of fibrous cap atheromata [54]) formed between FII^−/+^:ApoE^−/−^ (4 out of 10) and TM^Pro/Pro^:ApoE^−/−^ mice (10 out of 10) (n = 10 per group, p = 0.0108). In fact, the necrotic area within the lesions of the hypercoagulable mice was significantly increased: 56.2±10.8% of the total plaque area, as compared to 29.0±17.7% in the control ApoE^−/−^ group (n = 10 per group, p = 0.0024). (E) Hypocoagulable mice showed more stable advanced lesions, as indicated by the significantly thicker fibrous caps in comparison to ApoE^−/−^ mice (n = 10 per group, p = 0.0081). (F) Intima/media ratio was significantly increased in TM^Pro/Pro^:ApoE^−/−^ mice, whereas profoundly decreased in FII^−/+^:ApoE^−/−^ mice. Of note, the average outer diameter of the common carotid artery is 0.36 mm [21], thus suggesting that TM^Pro/Pro^:ApoE^−/−^ atherosclerotic plaques undergo a dramatic outward remodeling as indicated in panel (G). *p<0.05; **p<0.01; ***p<0.001. Error bars represent mean ± SD. Arrows indicate examples of positive staining/fibrous cap thickness. Abbreviations: H&E – hematoxylin and eosin; AL – advanced atherosclerotic lesion.

**Figure 3:**
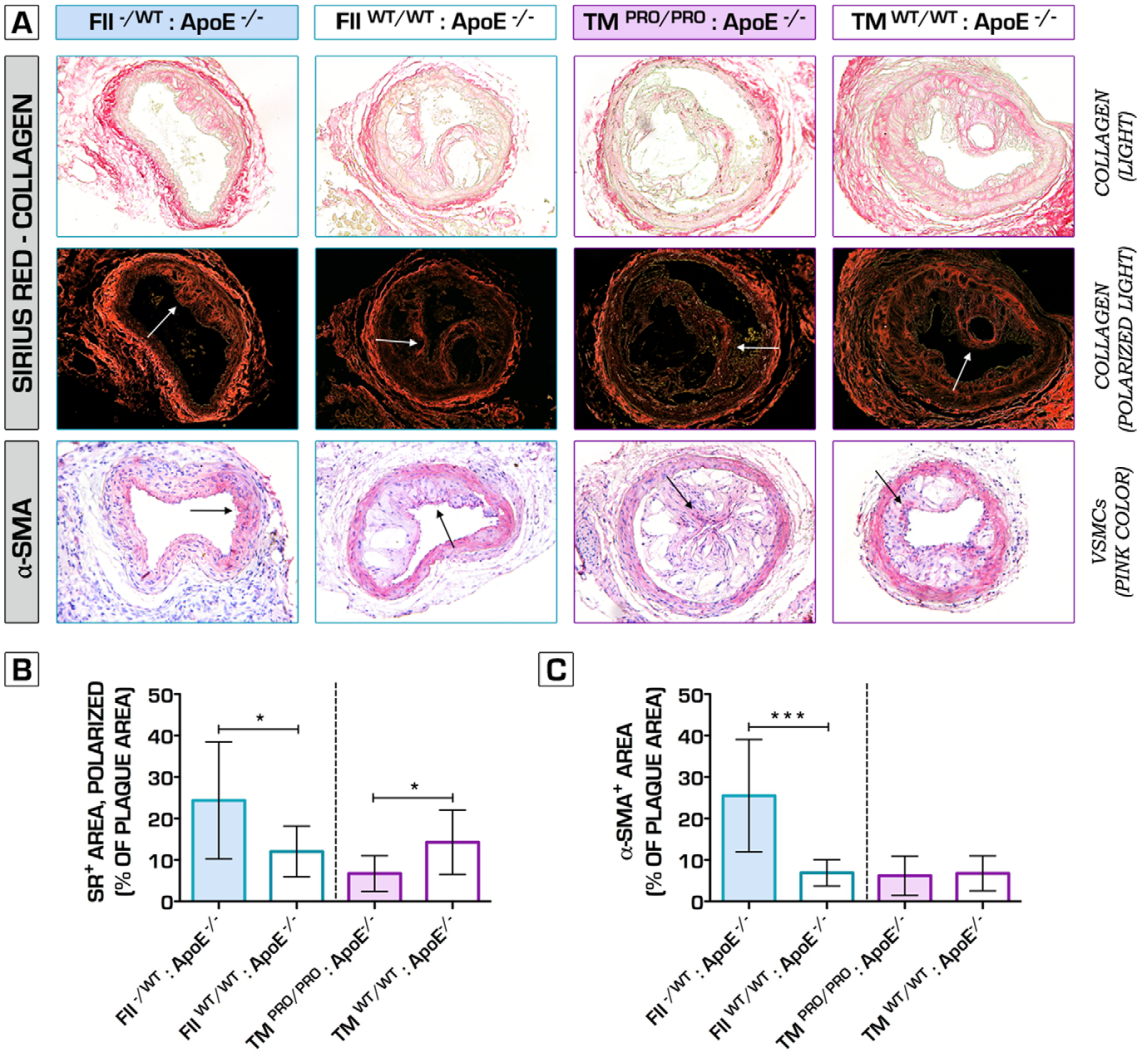
The role of hypo- and hypercoagulability in plaque fibrosis. Picrosirius red-stained sections assessed by light (**A, top row**) and polarized light (**A, second row**), indicate a significant decrease in the levels of collagen in TM^Pro/Pro^:ApoE^−/−^ carotid atherosclerotic plaques (6.7±4.3% vs. 14.3±7.8% of total plaque area in ApoE^−/−^ mice, n = 10 per group, p = 0.0193) (**B**). Hypocoagulable FII^−/+^:ApoE^−/−^ mice lesions showed a pro-fibrotic appearance, testified by increased collagen deposition (24.4±14.1% vs. 12.0±6.1% of total plaque area in ApoE^−/−^ mice, n = 10 per group, p = 0.0435) and α-smooth muscle actin content (25.5±13.6% vs. 6.9±3.2% of total plaque area in ApoE^−/−^ mice, n = 10 per group, p = 0.0003) (**B, C**). *p<0.05; **p<0.01; ***p<0.001. Error bars represent mean ± SD. Arrows indicate examples of positive staining. Abbreviations: SR – (Picro)sirius red; α-SMA - α-smooth muscle actin.

**Figure 4:**
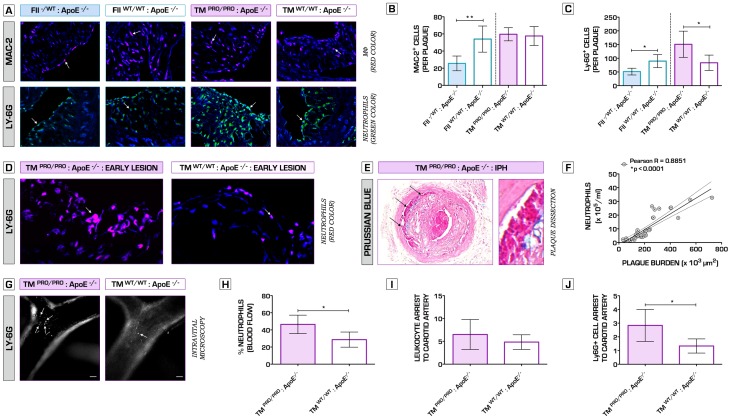
Hypercoagulability promotes neutrophil intraplaque recruitment and severe plaque phenotype. (A) Representative sections of atherosclerotic lesions formed in the carotid arteries of TM^Pro/Pro^:ApoE^−/−^ and FII^−/+^:ApoE^−/−^ mice (incl. control ApoE^−/−^ mice), stained for the presence of macrophages (MAC-2, red color, top row) and neutrophils (Ly-6G, green color, bottom row). Arrows show examples of positive cells. DNA is counterstained in blue. Macrophage and neutrophil infiltration were expressed as the absolute number of Mac-2^+^ and Ly-6G^+^ cells per plaque. (A, B) Hypocoagulability in FII^−/+^:ApoE^−/−^ mice promoted an anti-inflammatory plaque profile, indicated by a significant decrease in macrophage infiltration compared to control ApoE^−/−^ mice: 26±9 vs. 54±15 cells per plaque (n = 10 per group, p = 0.0067). No difference in macrophage content was observed between TM^Pro/Pro^:ApoE^−/−^ and ApoE^−/−^ mice atherosclerotic plaques: 60±8 vs. 58±11 cells per plaque (n = 10 per group, p = 0.7479). (A, C) Neutrophil accumulation was significantly increased in TM^Pro/Pro^:ApoE^−/−^ lesions (151±48 vs. 83±28 cells per plaque in ApoE^−/−^ mice, respectively; n = 10 per group; p = 0.0260). Interestingly, the opposite trend was observed in mice with genetically imposed hypoprothrombinemia (51±12 vs. 90±24 cells per plaque in ApoE^−/−^ mice, respectively; n = 10 per group; p = 0.0127). (D) Representative sections of early atherosclerotic lesions in external carotid artery of a TM^Pro/Pro^:ApoE^−/−^ mouse (left), abundantly infiltrated by Ly-6G^+^ cells (Ly-6G, red color), suggesting that hypercoagulability triggers lesion formation in a neutrophil-dependent manner. ApoE^−/−^ control mice are shown on the right hand side. (E) The panel represents an atherosclerotic dissection with superimposed thrombus formation in a TM^Pro/Pro^:ApoE^−/−^ mouse at 6 weeks after carotid collar placement. Using Perl’s Prussian blue stain (blue color), we detected free ferric ions deposited within the sites of plaque dissection, indicating the areas of intraplaque hemorrhage. Whereas the carotid lesions in 5 out of 10 TM^Pro/Pro^:ApoE^−/−^ mice were associated with either rupture, dissection or intraplaque hemorrhage, none of the control ApoE^−/−^ mice plaques had any signs of severe plaque vulnerability (Pearson's chi-squared test (*χ* ^2^), n = 10 per group, p = 0.0325). Statistical analysis including all experimental groups indicated that the number of circulating neutrophils in peripheral blood was strongly correlated to the extent of atherosclerotic plaque burden (F). Using intravital microscopy, we confirmed that the relative percentage of circulating neutrophils in TM^Pro/Pro^:ApoE^−/−^ mice was found significantly higher than ApoE^−/−^ control mice (n = 6 per group, p = 0.0107). Whereas there were no differences found in the general leukocyte rolling and arrest between TM^Pro/Pro^:ApoE^−/−^ and ApoE^−/−^ mice after 6 weeks on a high-fat diet (Rhodamine-labeled leukocytes) (n = 6 per group, p = 0.2886), Ly-6G^+^ neutrophils in TM^Pro/Pro^:ApoE^−/−^ mice were significantly more adherent to atherosclerotic lesions in the common carotid artery than in ApoE^−/−^ control mice (n = 6 per group, p = 0.0139). Bar represents 100 µm. (G,H,I, J). *p<0.05; **p<0.01; ***p<0.001. Error bars represent mean ± SD. Arrows indicate examples of positive staining. DNA was counterstained with Hoechst-33342 (blue). Abbreviations: MФ – Macrophages; IPH – intraplaque hemorrhage; TAT – thrombin-antithrombin complex.

**Figure 5:**
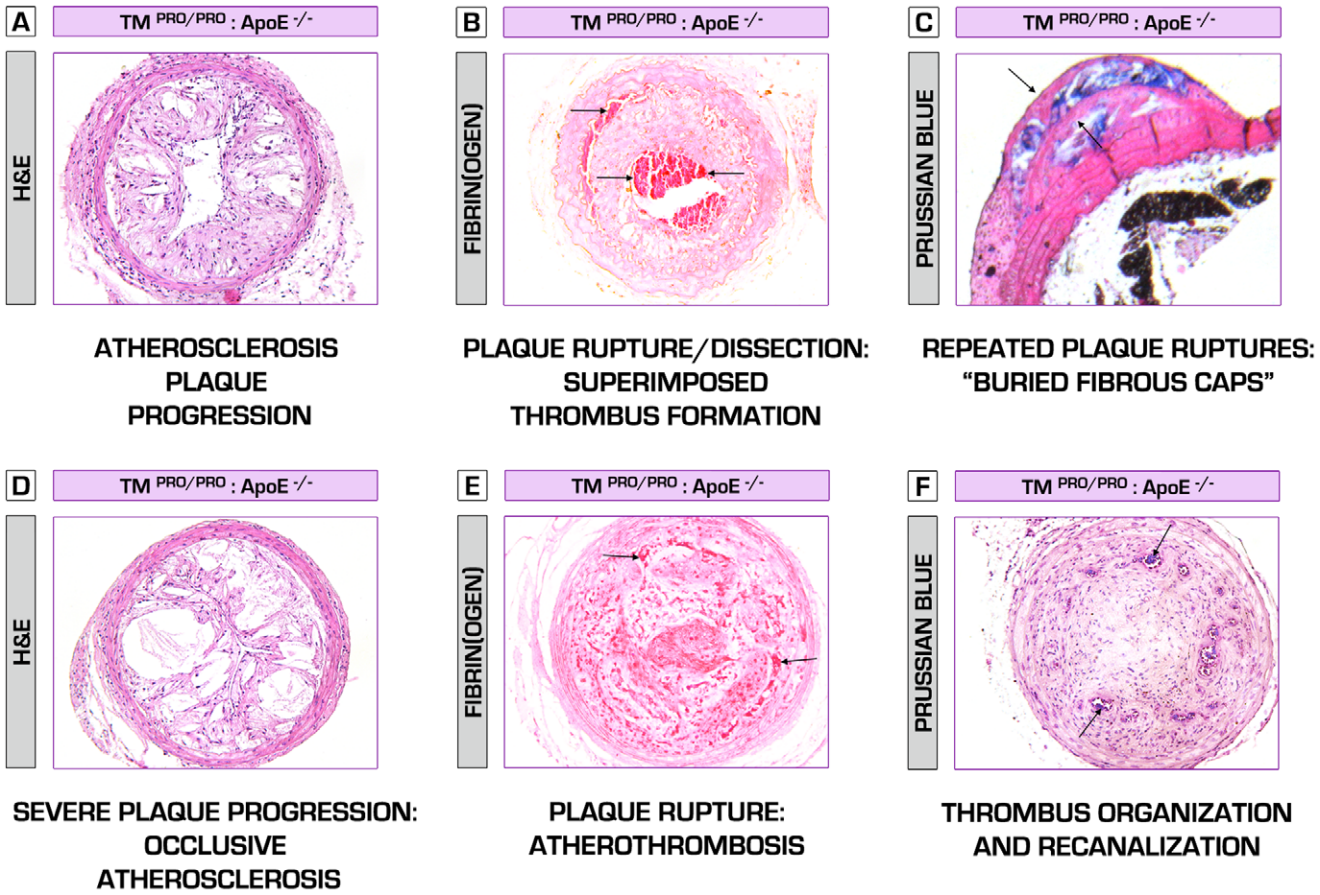
Hypercoagulable TM^Pro/Pro^:ApoE^−/−^ mice – a new mouse model of atherosclerotic plaque vulnerability. Here we present a new hypercoagulable atherosclerosis model, which closely mimics the composition and events leading to plaque destabilization, as normally observed in human atherothrombosis. In a series of sections, demonstrating carotid atherosclerotic plaques, obtained from TM^Pro/Pro^:ApoE^−/−^ mice at 6 weeks after collar placement on high-fat diet regimen, we show multiple signs of plaque vulnerability. (A) A non-occlusive but rapidly progressing atherosclerotic lesion, characterized by abundant infiltration of leukocytes. (B) TM^Pro/Pro^:ApoE^−/−^ mice plaques tend to rupture and dissect (upper arrow) even during the non-occlusive phase, accompanied by “silent” intraluminal thrombosis (lower arrows). Despite the detrimental pathologic characteristics of those lesions, these data confirm the hypothesis that arterial thrombosis might exist long before a fatal event takes place. This is further consolidated by the presence of so called “buried fibrous caps” (indicated by the arrows) in TM^Pro/Pro^:ApoE^−/−^ mice plaques [55], considered a marker of healed plaque ruptures, and also observed in human atherosclerosis. Blue color denotes a massive intraplaque hemorrhage (iron ions deposition) (C). Hypercoagulability induces a severe inflammatory and pro-necrotic intraplaque environment, leading to the formation of enormous necrotic core, thin fibrous caps, further plaque destabilization (D) and atherothrombosis (occlusive intraluminal thrombosis/abundant fibrin(ogen) deposition (indicated by the arrows)) (E). Thrombi undergo fibrotic organization involving vascular smooth muscle cells and fibroblasts ingrowth, and are then partially recanalized by newly formed vessels (arrows, blue color – iron deposition/presence of erythrocytes)(F).

Hence, to further verify the net effects of underlying alterations in clotting potential on plaque phenotype, we also studied the impact of both genetic perturbations on collar-induced carotid artery atherosclerosis [21]. High-fat diet fed FII^−/+^:ApoE^−/−^ mice displayed significantly decreased plaque volume, degree of stenosis, intima/media ratio and expansion of the arterial wall, 6 weeks after bilateral perivascular carotid collar placement (*Figure 2A,B,C,F,G*). Furthermore, hypocoagulability ameliorated plaque stability, testified by a significantly increased mean fibrous cap thickness (*Figure 2A,E*). Conversely, TM^Pro/Pro^:ApoE^−/−^ mice lesions were substantially larger, accompanied by significantly increased luminal stenosis, intima/media ratio and outward remodeling (*Figure 2A,B,C,F,G*). Similar to spontaneous atherosclerosis, collar-induced carotid artery plaques in FII^−/+^:ApoE^−/−^ mice presented a stable pro-fibrotic phenotype, whereas TM^Pro/Pro^:ApoE^−/−^ lesions showed pronounced features of plaque vulnerability, including larger necrotic cores (*Figure 2A,D*), thin fibrous caps (*Figure 2A,E*) and significant decrease in collagen content (*Figure 3A,B*). Immunofluorescence microcopy analyses for caspase-3 revealed enhanced apoptosis within the lesions of the procoagulant ApoE^−/−^ mice (data not shown). High plaque vulnerability in hypercoagulable mice was also associated with a pro-inflammatory plaque phenotype, signs of intraplaque hemorrhage, plaque dissection, but also spontaneous atherothrombosis (*Figure 4E* *,* *Figure 5*), whereas fibrin deposits were extensively distributed throughout ruptured TM^Pro/Pro^:ApoE^−/−^ lesions (*Figure 5*). Intraplaque accumulation of macrophages and neutrophils was significantly diminished in hypocoagulable mice (*Figure 4A,B,C*), indicating that deficiency in prothrombin results in a less inflammatory plaque profile. In contrast, in TM^Pro/Pro^:ApoE^−/−^ advanced carotid artery lesions were abundantly infiltrated with neutrophils when compared to ApoE^−/−^ control mice, whereas no changes were observed in terms of macrophage content (*Figure 4A,B,C*). A pronounced Ly6G^+^ cell intraplaque recruitment was also observed during early stages of atherosclerosis in TM^Pro/Pro^:ApoE^−/−^ mice (*Figure 4D*). To provide further understanding into how alterations in blood coagulation potential affect the thrombogenicity of the arterial vessel wall, we pursued complementary studies of vessel occlusion following ferric chloride injury of healthy arteries. Times to occlusion and cessation of blood flow as a result of thrombus formation were significantly shortened in TM^Pro/Pro^:ApoE^−/−^ mice relative to control ApoE^−/−^ mice, whereas occlusion times in FII^−/+^:ApoE^−/−^ animals was comparable to control ApoE^−/−^ cohorts (*Figure S2*).

### Hypercoagulability Triggers Initiation and Progression of Atherosclerotic Lesions in a Neutrophil-Dependent Manner

There was a strong positive association between the number of circulating neutrophils in peripheral blood and the extent of atherosclerotic plaque burden (*Figure 4F*). No significant correlation was observed between the number of peripheral blood monocytes and plaque size. Because of the increased neutrophil counts observed in hypercoagulable TM^Pro/Pro^:ApoE^−/−^ mice after 35 weeks on a regular chow diet (*Table S1C*), and the abundant infiltration of neutrophils within vulnerable-appearing atherosclerotic lesions (*Figure 4A,C*), we explored the impact of hypercoagulability on neutrophil function and hematopoiesis in the context of atherosclerosis. Intravital microscopy studies revealed that neutrophils were significantly more adherent to atherosclerotic lesions in the common carotid artery of TM^Pro/Pro^:ApoE^−/−^ than in ApoE^−/−^ control mice after 6 weeks on a high-fat diet (*Figure 4G,H,I,J*). These data consolidated our histological findings (*Figure 4A,C,D*), suggesting that hypercoagulability can promote initiation and progression of atherosclerotic lesions in a neutrophil-dependent manner.

### Hypercoagulability and Its Effects on Systemic Inflammation and Hematopoiesis: Enhanced Accumulation of Reactive Oxygen Species in Neutrophils

**Figure 6:**
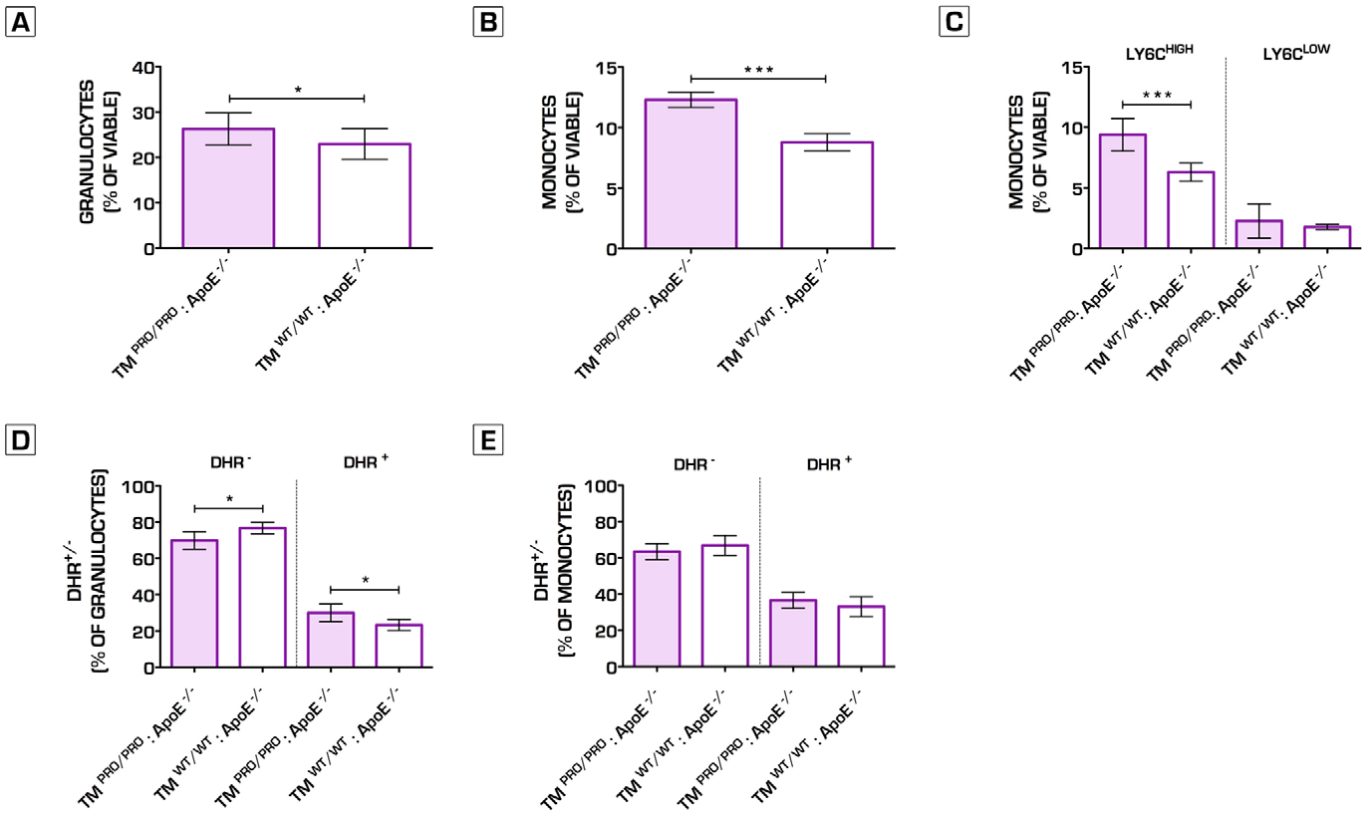
Hypercoagulability induces oxidative stress in granulocytes within the bone marrow compartment. Granulocytes and monocytes cell fractions in the bone marrow were significantly increased in TM^Pro/Pro^:ApoE^−/−^ as compared to ApoE^−/−^ control mice after 8 weeks on a regular chow diet (Granulocytes: 26.3±3.6% vs. 22.9±3.4%; n = 12 per group, p = 0.0292)(Monocytes: 12.3±0.6% vs. 8.8±0.7%; n = 12 per group, p<0.0001) (A, B). The significant increase in monocytes can be explained by the higher relative numbers of Ly6C^HIGH^ monocyte cells in TM^Pro/Pro^:ApoE^−/−^ mice bone marrow (Ly6C^HIGH^ cells: 9.4±1.3% vs. 6.3±0.8%; n = 12 per group, p = 0.0002) (C). Using DHR123 FACS analysis, we analyzed the amount of oxidative burst activity in granulocytes and monocytes in the bone marrow after PMA stimulation. The monocytes did not show any differences in DHR signal and thus ROS activity, whereas in the granulocytes of the TM^Pro/Pro^:ApoE^−/−^ mice, a significant increase was observed in the DHR signal when compared to ApoE^−/−^ mice, indicating enhanced oxidative stress upon PMA stimulation in the TM^Pro/Pro^:ApoE^−/−^ granulocytes present in the bone marrow (D, E). *p<0.05; **p<0.01; ***p<0.001. Error bars represent mean ± SD. Abbreviations: DHR123– Dihydrorhodamine 123; ROS – Reactive Oxygen Species; PMA - Phorbol 12-Myristate 13-Acetate.

Consistent with this view, hypercoagulability promoted a significant increase in plasma CCL2 and CXCL1 levels (*Table S2*). TM^Pro/Pro^:ApoE^−/−^ mice showed significantly higher IL-6 plasma levels after 35 weeks on regular chow diet. Although there were trends toward increased IL-1β, expression of other key pro-inflammatory cytokines such as TNF-α, IFN-γ, IL-5 and IL-12 were not statistically different between hypercoagulable and control ApoE^−/−^ mice, indicating that TM^Pro/Pro^:ApoE^−/−^ mice exhibited a pro- but not hyper-inflammatory systemic profile (*Table S2*). In addition, the higher plasma expression levels of granulocyte-colony stimulating factor (G-CSF) in TM^Pro/Pro^:ApoE^−/−^ mice raises the possibility that the loss of TM function not only impacts on thrombin activity but also affects granulopoiesis in the bone marrow. However, we did not detect major changes in hematopoiesis between hypercoagulable and control ApoE^−/−^ mice after 10 weeks on regular chow diet (*Figure S3*), including any preferential differentiation towards granulocytic-type colonies. Despite a minor but significant increase in the common myeloid progenitor (CMP) cells, lineage-negative (LK, LS, LSK), granulocyte-macrophage progenitor (GMP) and erythroid/megakaryocyte progenitor (EMP) populations in TM^Pro/Pro^:ApoE^−/−^ mice remained unaffected (*Figure S3*). Nevertheless, the relative percentage of mature granulocytes in bone marrow, as well as of pro-atherogenic Ly6C^high^ monocytes [22] as measured by flow cytometry, was significantly increased in the pro-thrombotic mice (*Figure 6A,B,C*). Of interest, another consequence of chronic hypercoagulability was enhanced accumulation of reactive oxygen species in neutrophils and not monocytes, as assessed by DHR fluorescence, the latter considered a measure of neutrophil senescence (*Figure 6D,E*).

### Administration of Direct Thrombin Inhibitor Dabigatran Etexilate (DE) or rmAPC Substantially Decreases Systemic Inflammation, Aborts Atherosclerosis, Promotes Plaque Stability And Prevents Against Atherothrombosis in Hypercoagulable Mice

**Figure 7:**
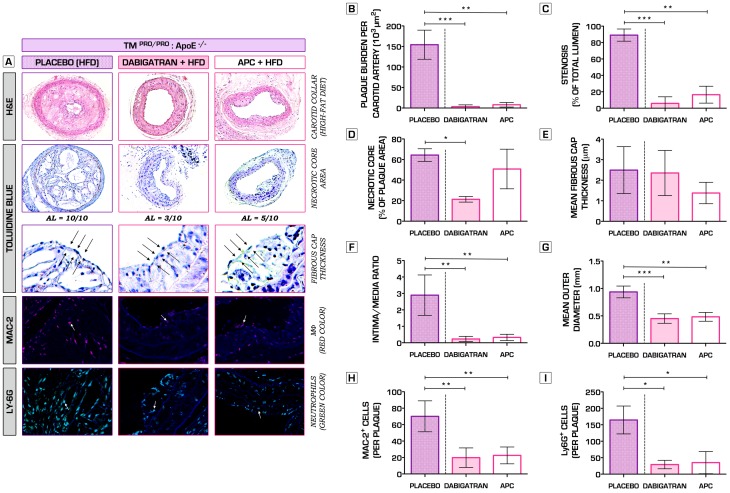
Inhibition of thrombin activity by administration of direct thrombin inhibitor Dabigatran etexilate or recombinant murine APC substantially attenuates leukocyte recruitment and prevents against severe atherosclerosis progression and atherothrombosis. (A) Representative hematoxylin and eosin (H&E)-stained sections of atherosclerotic lesions formed in carotid arteries of TM^Pro/Pro^:ApoE^−/−^ mice, which were assigned to different intervention arms (oral Dabigatran etexilate - 7.5 mg DE/gram chow; i.p. administered bolus doses of recombinant murine APC - 2.5 mg/kg/per every 5 days; or placebo) for a total of 6 weeks after cuff placement around the common carotid arteries (top row). Toluidine blue (TB) stainings were used to quantify the size of necrotic core areas (second and third row). Whereas placebo treated TM^Pro/Pro^:ApoE^−/−^ mice all developed advanced lesions (identified by the presence of necrotic core and fibrous cap formation), Dabigatran etexilate- (3 out of 10, Pearson's chi-squared test (*χ* ^2^), n = 10 per group, p = 0.0031 vs. placebo) and rAPC-treated mice (5 out of 10, Pearson's chi-squared test (*χ* ^2^), n = 10 per group, p = 0.0325 vs. placebo) had significantly reduced atheromata formed. A total of 5 out 10 animals in the placebo group showed signs of severe plaque vulnerability, whereas none were observed in the intervention arms. Atherosclerotic plaques were further analyzed for the presence of macrophages (MAC-2, red color, fourth row) and neutrophils (Ly-6G, green color, bottom row). Arrows show examples of positive cells. Macrophage and neutrophil infiltration were expressed as the absolute number of Mac-2^+^ and Ly-6G^+^ cells per plaque. (B) Administration of either Dabigatran etexilate or rAPC rescued the phenotype and pronouncedly reduced atherosclerotic plaque burden (Placebo: 154.3±35.5*10^3^ µm^2^; Dabigatran Etexilate: 3.3±4.4*10^3^ µm^2^, p<0.001; rAPC: 7.9±5.5*10^3^ µm^2^, p<0.01; n = 10 per group). (C, F, G) These findings were further consolidated by a significant decrease in the degree of stenosis (with ∼80%), intima/media ratio and outward remodeling within the treatment arms of the study (n = 10 per group). (D, E) Except for a significant reduction of the necrotic core area in the Dabigatran etexilate-treated mice as compared to placebo group (n = 10 per group, p<0.05), no other effects were observed with regard to necrotic core formation or fibrous cap thickness. Of note, only mice having advanced lesions were included in these analyses (Dabigatran etexilate: n = 3; rAPC: n = 5). (H, I) In addition, TM^Pro/Pro^:ApoE^−/−^ mice treated with direct thrombin inhibitor or natural anticoagulant rAPC developed an anti-inflammatory stable plaque phenotype, associated with substantially reduced levels of macrophage and neutrophil recruitment. *p<0.05; **p<0.01; ***p<0.001. Error bars represent mean ± SD. Arrows indicate examples of positive staining/fibrous cap thickness. DNA was counterstained with Hoechst-33342 (blue). Abbreviations: HFD – high-fat diet; AL – advanced atherosclerotic lesion; MФ- macrophage; rAPC – recombinant murine activated protein C.

**Figure 8:**
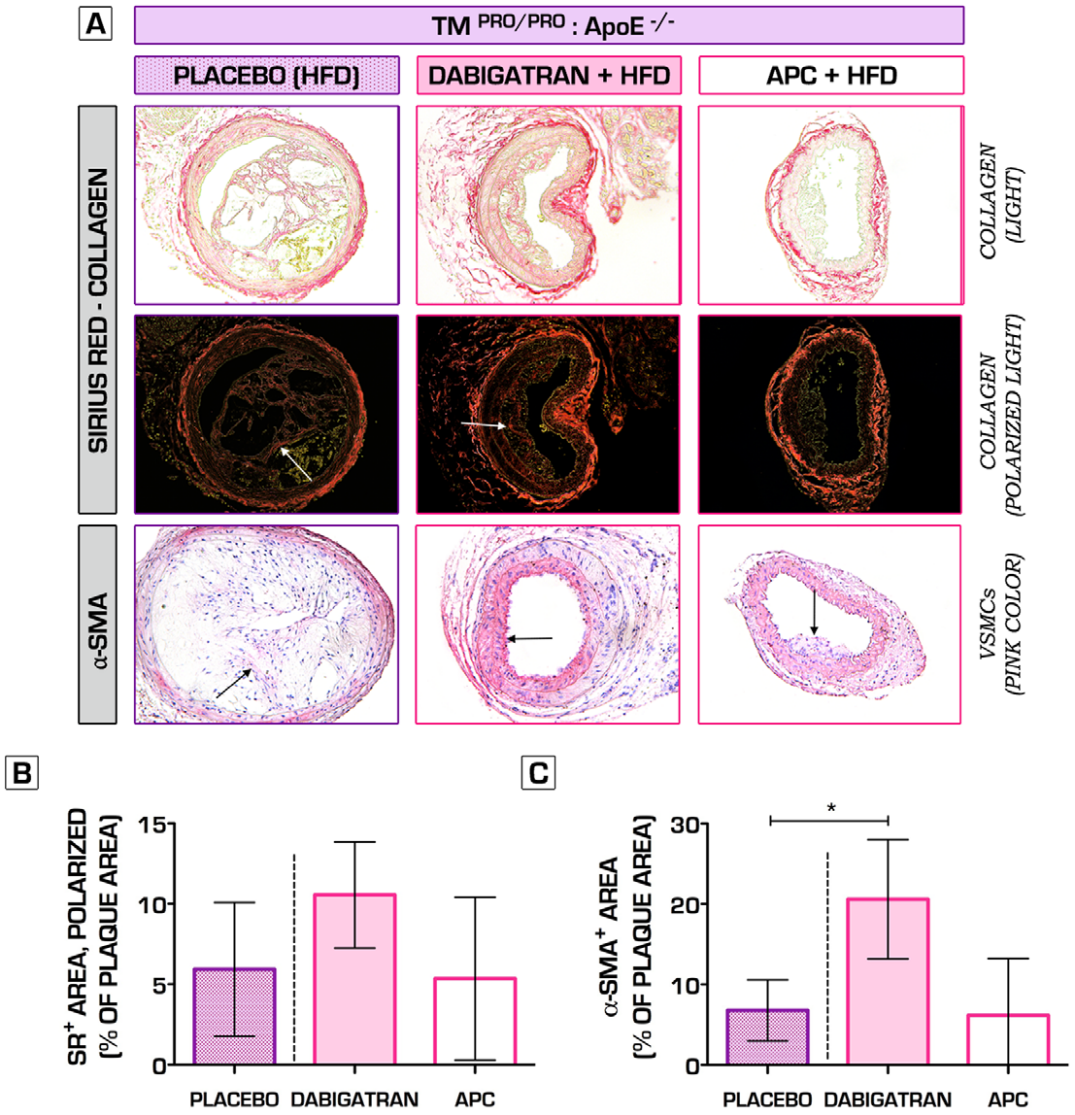
The effects of direct and indirect inhibition of thrombin activity on plaque fibrosis. Picrosirius red-stained sections assessed by light (A, top row) and polarized light (A, second row), indicate no significant changes in collagen content in TM^Pro/Pro^:ApoE^−/−^ mice after 6-week treatment with either Dabigatran etexilate or mouse rAPC, as compared to placebo (B). Administration of oral Dabigatran etexilate led to a significant increase in α-smooth muscle actin intraplaque content in TM^Pro/Pro^:ApoE^−/−^ mice vs. placebo treatment (20.6±7.4% vs. 6.8±3.8% of total plaque area, n = 10 per group, p<0.05) (A, C). rAPC therapy did not have an effect (6.2±7.1% of total plaque area, n = 10 per group, p>0.05) (A, C). *p<0.05; **p<0.01; ***p<0.001. Error bars represent mean ± SD. Arrows indicate examples of positive staining. Abbreviations: SR – (Picro)sirius red; α-SMA - α-smooth muscle actin; HFD – high-fat diet; VSMC – vascular smooth muscle cells; rAPC – recombinant mouse activated protein C.

To study the role of thrombin in modulating atherogenesis *in vivo*, we administered either the specific oral thrombin inhibitor DE or a recombinant form of the natural anti-coagulant APC for 6 weeks after carotid collar placement in hypercoagulable TM^Pro/Pro^:ApoE^−/−^ mice on high-fat diet. Remarkably, both interventions completely rescued plaque formation (*Figure 7A,B*), as also evident by the decreased degree of stenosis, intima/media ratio and positive outward remodeling (*Figure 7C,F,G*). Whereas in the placebo group 5 out of 10 animals had plaques with overt signs of plaque vulnerability (defined as, i.e. plaque dissection, intraplaque hemorrhage or superimposed thrombus formation), oral DE or rmAPC treatments limited the occurrence of plaque destabilization and atherothrombotic phenomena, and resulted in substantially reduced leukocyte recruitment and enhanced plaque stability (*Figure 7A,H,I* *;* *Figure 8*). In addition, both interventions led to a pronounced decrease in thrombin generation, suggesting that even ApoE^−/−^ mice exerted a low-grade hypercoagulable state (*Table S1A, Table S3A*). DE and rmAPC therapies significantly limited systemic inflammation (*Table S3C*), as further exemplified by decreased neutrophil and lymphocyte counts and cytokine and chemokine profiles that show a shift to an anti-inflammatory state (*Table S4*).

## Discussion

### Major Findings

These studies provide strong evidence directly documenting that thrombin and other hemostatic system components are powerful determinants of inflammatory vessel wall disease, and even capable of superseding other pro-atherosclerotic insults. We here demonstrate that thrombin activity can influence onset, progression and qualitative properties of atherosclerotic plaques. In two distinct experimental setups (spontaneous and collar-induced atherosclerosis), we show that genetically-imposed 50% reduction in prothrombin (FII^−/+^) in atherosclerosis-prone ApoE^−/−^ mice remarkably diminishes lesion formation and promotes plaque stability. In contrast, mice with genetically impaired anticoagulant function of TM, crossed on ApoE^−/−^ background, develop severe atherosclerotic disease. We here for the first time demonstrate the importance of neutrophils in the coagulation-inflammation interplay during atherogenesis. The principal finding of this study is that hypercoagulability induces enhanced mobilization of neutrophils from the bone marrow into the circulation, accompanied with neutrophil hyper-reactivity, increased oxidative stress, apoptosis and abundant intraplaque neutrophil infiltration, thus promoting unstable atherosclerotic plaque phenotype and spontaneous atherothrombosis. Administration of either the synthetic specific thrombin inhibitor DE or a recombinant form of the natural anticoagulant APC, counteract the pro-inflammatory, pro-atherogenic and pro-thrombotic phenotype of hypercoagulable TM^Pro/Pro^:ApoE^−/−^ mice, resulting in plaque stability and preventing atherothrombosis.

### Coagulation and Inflammation in Atherosclerosis

Given the multifactorial nature of atherosclerosis and the well-known capacity of coagulation proteases and their receptors (protease-activated receptors, PARs) and substrates to control inflammatory and reparative processes [18], one would anticipate that hemostatic factors might contribute, at least incrementally, to plaque development. In fact, various pro-thrombotic states have been associated with enhanced atherosclerosis progression in mice *in vivo* [23]–[29]. Nevertheless, the mechanisms through which clotting contributes to atherosclerosis progression remain unclear to date. Thrombin is a central coagulation protease, which through the activation of PAR-1 is known to promote numerous pro-atherogenic actions *in vitro* such as endothelial permeability, migration and proliferation of VSMC, platelet activation, leukocyte adhesion and recruitment, cytokine and chemokine production, vascular calcification, angiogenesis and apoptosis [13]. Similar effects can also be triggered via both PAR-1 and PAR-2 by either TF-FVIIa complex or FXa [18]. In contrast, APC counteracts inflammation through PAR-1 signaling at multiple levels such as enhancing the endothelial barrier integrity, attenuating TF and TNF-α release by monocytes, inhibiting cytokine production, leukocyte endothelial transmigration and NF-κB pathways [30], [31].

Our data strongly suggests that increased thrombin generation due to diminished APC production in TM^Pro/Pro^:ApoE^−/−^ mice may be mechanistically-coupled to the pro-atherosclerotic phenotype. Previous studies have indicated that hypercoagulability can have beneficial effects on plaque stenosis during the intermediate phases of progression by promoting positive vascular remodeling [17]. Importantly, our data shows that plaques of prothrombotic mice had profound composition changes, with overt features of plaque vulnerability at later stages of disease development (at 35 weeks on regular chow diet), characterized by the presence of large necrotic cores, thin fibrous caps, significantly increased neutrophil intraplaque infiltration and apoptosis, decreased collagen and VSMC content, occlusive stenosis and spontaneous atherothrombosis. Despite that we show a significant association between hypercoagulability and high rate of spontaneous mortality in TM^Pro/Pro^:ApoE^−/−^ mice, further studies are needed to precisely determine the underlying cause of this observation.

There are numerous pathways, which have been implicated to play a role in the complex interplay between coagulation and inflammation in various pathologic conditions [30]. Here, for the first time, we show the strong potential of the coagulation system (in particular thrombin) to regulate inflammation in atherosclerosis as highlighted by the effects of direct thrombin inhibition therapy on leukocyte counts, chemokine and cytokine levels. Neutrophils represent another intriguing cellular interface between blood coagulation and inflammation [3], [32]. Although the importance of neutrophils in atherosclerosis remains to be defined in detail, several studies have highlighted their pro-atherogenic potential and proposed role in atherosclerotic plaque destabilization [18], [33]–[38]. Our data demonstrate that hypercoagulability promotes enhanced accumulation of reactive oxygen species in neutrophils and, thus triggering enhanced neutrophil senescence. Systemic inflammation involving activated neutrophils is associated with unstable coronary artery disease and considered an independent predictor for cardiovascular outcome in patients [39]. Our data indicate increased neutrophil rolling and arrest on early carotid atherosclerotic plaques in TM^PRO/PRO^:ApoE^−/−^ mice. In addition, the significantly higher neutrophil intraplaque infiltration, consolidated by striking correlations between neutrophil counts and the amount of plaque burden, suggest a key role for neutrophils in this coagulation-inflammation interplay in atherosclerosis. The increased number of circulating pro-atherogenic neutrophils in TM^Pro/Pro^:ApoE^−/−^ mice can be in part explained by enhanced mobilization from the bone marrow as a result of exuberant plasma G-CSF, CCL-2 and CXCL-1 expression. G-CSF is an essential regulator of the neutrophil mobilization from the bone marrow. Numerous experimental and human studies have shown an association between higher G-CSF plasma levels, neutrophil activation, endothelial dysfunction, enhanced oxidative stress, hypercoagulability and platelet aggregation [40]–[42]. CCL-2 and CXCL-1 are chemokines recognized for their potent neutrophil chemoattractant activity and capacity to promote vascular inflammation [32], but also known to be critical players in recruitment of monocytes and neutrophils to sites of chronic inflammation [39], [43], [44]. Thrombin acts as a secretagogue, promotes endothelial dysfunction and induces the release of P-selectin, which is stored in the Weibel-Palade bodies [45]. P-selectin is a powerful mediator of neutrophil adhesion to the endothelium, but also plays an important role in atherogenesis [29], [45]. Thrombin is among one of the most potent platelet activators [18]. One may also assume that part of the pro-atherogenic effects observed in the hypercoagulable mice can be also mediated via platelet activation, known for their crucial role in atherosclerosis progression [46], [47]. Platelets interact with a variety of cellular partners such as monocytes, neutrophils, endothelial cells, endothelial progenitor cells, and others, thus induce key inflammatory responses including leukocyte adhesion, migration, proteolysis, thrombosis, but also facilitate the differentiation of macrophages to foam cells [48]. Although we did neither detect any significant correlation between the number of peripheral monocytes and atherosclerotic plaque burden, nor changes in the number of macrophages infiltrating the lesions in TM^PRO/PRO^:ApoE^−/−^ versus control mice, this also does not rule out a role of monocytes in atherogenesis. Neutrophils can rapidly undergo apoptosis as a result of the enhanced oxidative stress. The abundant number of pro-apoptotic leukocytes within the lesions of TM^PRO/PRO^:ApoE^−/−^ mice can overload the phagocytic clearance capacity of the macrophages, thus promoting enhanced macrophage death and subsequent plaque necrosis [49]. In fact, the necrotic core areas and apoptotic indices within the lesions of hypercoagulable mice were substantially increased.

### Conclusions and Potential Clinical Implications

Steadily increasing thrombin-antithrombin plasma levels, considered a sensitive marker of thrombin formation *in vivo*, were independently associated with the presence and severity of coronary atherosclerotic plaques, as defined by coronary computed tomographic angiography (CCTA) [50]. In conclusion, we here provide substantial new evidence showing that controlling coagulation via thrombin inhibition is a potential new therapeutic target to treat atherosclerosis. Given the promising safety profile and significant clinical benefits, which selective anticoagulants may offer over traditional anticoagulant therapy [51], [52], including the reduction of risk of stroke and all-cause mortality after acute coronary syndromes, the potential clinical importance of these findings allows the unique opportunity to study if and how administration of novel classes of anticoagulants modifies atherosclerosis phenotype in patients. Nevertheless, due to the large heterogeneity in humans, the multifactorial nature of atherosclerosis, the dual-faceted character that many coagulation factors can exert, and their beneficial roles under normal physiological conditions, one should consider long-term specific coagulation inhibition with caution [53]. There is an urgent need of clinical trials to fully assess the overall benefit and risk balance of long-term therapy with novel oral anticoagulant agents.

## Supporting Information

Figure S1 **Hypercoagulability in TM^Pro/Pro^:ApoE^−/−^ mice does not alter lipid uptake in bone marrow-derived macrophages (BMM).** (A) There were no significant differences found in the lipid uptake in BMM derived from TM^Pro/Pro^:ApoE^−/−^ and control ApoE^−/−^ mice, as determined by flow cytometry analysis. (B, C, D) In addition, we also used high performance thin layer chromatography to test the free cholesterol, cholesterol esters and triglycerides accumulation in BMM in response to LDL and oxidized LDL loading and there were no significant differences detected between BMM obtained from TM^Pro/Pro^:ApoE^−/−^ and control ApoE^−/−^ mice. Error bars represent mean ± SD. Abbreviations: HP-TLC - high performance thin layer chromatography; BMM - Bone marrow-derived macrophages; LDL – low-density lipoprotein; oxLDL – oxidized low-density lipoprotein.
(DOC)

Figure S2 **20% FeCl_3_-induced arterial injury in hyper- and hypocoagulable atherosclerosis-prone mice.** Time to occlusion (TTO) and closing times (CT) were established. TTO is defined as the time after FeCl_3_ application required for the blood flow to decline to 90%, whereas CT represents the time from the start of flow reduction to a complete occlusion of the carotid artery. (A, B) Both TTO and CT were significantly shortened in TM^Pro/Pro^:ApoE^−/−^ as compared to ApoE^−/−^ control mice (TTO: 4.4±0.9 vs. 14.1±11.1 min., respectively; n = 10 per group, p = 0.0010) (CT: 1.2±0.8 vs. 11.3±13.0 min., respectively; n = 10 per group, p = 0.0010), suggesting for a pro-thrombotic arterial vessel wall phenotype. In contrast, hypocoagulability in FII^−/+^:ApoE^−/−^ mice had no effect on thrombus formation during FeCl_3_-induced arterial injury. Of note, all 10 out of 10 of the TM^Pro/Pro^:ApoE^−/−^ mice formed an occlusive thrombus (animals depicted at 30 min. represent all mice, which did not induce occlusive thrombus formation, indicated by an arrow). *p<0.05; **p<0.01; ***p<0.001. Dotted lines represent mean.
(DOC)

Figure S3 **The effects of hypercoagulability on hematopoiesis.** Using a CFU-C (colony forming unit in culture) assay, we established that there were no significant differences in the amount of total colonies produced by TM^Pro/Pro^:ApoE^−/−^ as compared to ApoE^−/−^ control mice after 8 weeks on a regular chow diet (A). Furthermore, we could not find any changes in the composition, as determined by the CFU subset analysis, indicating that hypercoagulability does not affect hematopoiesis in the bone marrow compartment (B). FACS analysis of the bone marrow consolidated the results of the CFU-C assay (C, D, E). The amount of LSK (Lin−/Sca-1+/c-Kit+) cells showed a tendency towards an increase in the TM^Pro/Pro^:ApoE^−/−^ compared to ApoE^−/−^ control mice (4.2±0.8% vs. 3.7±0.7%; n = 12 per group, p = 0.0529) (F). The amount of CMP (common myeloid progenitor) cells was significantly increased in the TM^Pro/Pro^:ApoE^−/−^ mice compared to the controls (15.1±3.3% vs. 12.7±2.3%; n = 12 per group, p = 0.0402). (G). In addition, EMP and GMP populations in the bone marrow remained unaffected by the hypercoagulable state in TM^Pro/Pro^:ApoE^−/−^ mice (H, I). *p<0.05; **p<0.01; ***p<0.001. Error bars represent mean ± SD. Abbreviations: CFU - colony forming unit; GM - granulocyte-macrophage progenitor; G - granulocyte progenitor; M - macrophage progenitor; LK - cells positive for LIN^−^c-Kit^+^Sca-1^−^ lineage markers; LSK - cells positive for LIN^−^c-Kit^+^Sca-1^+^ lineage markers; CMPs - common myeloid progenitors; GMP - granulocyte/macrophage progenitors; EMP – erythroid/megakaryocyte progenitors.
(DOC)

Table S1 **Coagulation profile (A), body weight, lipid profile (B) and complete blood counts (C), assessed after 35 weeks on regular chow diet in FII^−/+^:ApoE^−/−^, TM^Pro/Pro^:ApoE^−/−^ and control ApoE^−/−^ mice (n = 10 per group).** *p<0.05; **p<0.01; ***p<0.001. Data are presented as mean ± SD. Abbreviations: ETP – Endogenous Thrombin Potential; TAT – Thrombin-Antithrombin Complex; HDL – High-Density Lipoprotein; LDL – Low-Density Lipoprotein; CBC – Complete Blood Count; RBC – Red Blood Cells; WBC – White Blood Cells; Hgb – Hemoglobin; Hct – Hematocrit.
(DOC)

Table S2 **Cytokine and chemokine profile assessed after 35 weeks on regular chow diet in FII^−/+^:ApoE^−/−^, TM^Pro/Pro^:ApoE^−/−^ and control ApoE^−/−^ mice (n = 10 per group).** *p<0.05; **p<0.01; ***p<0.001. Data are presented as mean ± SD. Abbreviations: IL – interleukin; TNF-α - tumor necrosis factor-alpha; IFN-γ - Interferon-gamma; G-CSF - Granulocyte colony-stimulating factor; MCP-1 - monocyte chemotactic protein-1; MIP-1α - Macrophage inflammatory protein-1α; MIP-1β - Macrophage inflammatory protein-1β; RANTES - Regulated upon Activation, Normal T-cell Expressed, and Secreted; KC - keratinocyte chemoattractant.
(DOC)

Table S3 **Coagulation profile (A), lipid profile (B) and complete blood counts (C), assessed at 6 weeks after carotid collar placement in TM^Pro/Pro^:ApoE^−/−^ on high-fat diet and treated with placebo, oral Dabigatran etexilate or mouse recombinant APC (n = 10 per group).** *p<0.05; **p<0.01; ***p<0.001 (Intervention groups compared to placebo group). Data are presented as mean ± SD. Abbreviations: TAT – Thrombin-Antithrombin Complex; HDL – High-Density Lipoprotein; LDL – Low-Density Lipoprotein; CBC – Complete Blood Count; RBC – Red Blood Cells; WBC – White Blood Cells; Hgb – Hemoglobin; Hct – Hematocrit; APC – Activated Protein C.
(DOC)

Table S4 **Cytokine and chemokine profile assessed at 6 weeks after carotid collar placement in TM^Pro/Pro^:ApoE^−/−^ on high-fat diet and treated with placebo, oral Dabigatran etexilate or mouse recombinant APC (n = 10 per group).** *p<0.05; **p<0.01; ***p<0.001 (Intervention groups compared to placebo group). Data are presented as mean ± SD. Abbreviations: IL – interleukin; TNF-α - tumor necrosis factor-alpha; IFN-γ - Interferon-gamma; G-CSF - Granulocyte colony-stimulating factor; MCP-1 - monocyte chemotactic protein-1; MIP-1α - Macrophage inflammatory protein-1α; MIP-1β - Macrophage inflammatory protein-1β; RANTES - Regulated upon Activation, Normal T-cell Expressed, and Secreted; KC - keratinocyte chemoattractant; APC – Activated Protein C.
(DOC)

Methods S1 (DOC)
