# Supplementary figures and images for: Genetic and Pharmacological Modifications of Thrombin Formation in Apolipoprotein E-deficient Mice Determine Atherosclerosis Severity and Atherothrombosis Onset in a Neutrophil-Dependent Manner
Source: PLoS One. 2013 Feb 7;8(2):e55784. doi: 10.1371/journal.pone.0055784 (PMC3567111; doi:10.1371/journal.pone.0055784)

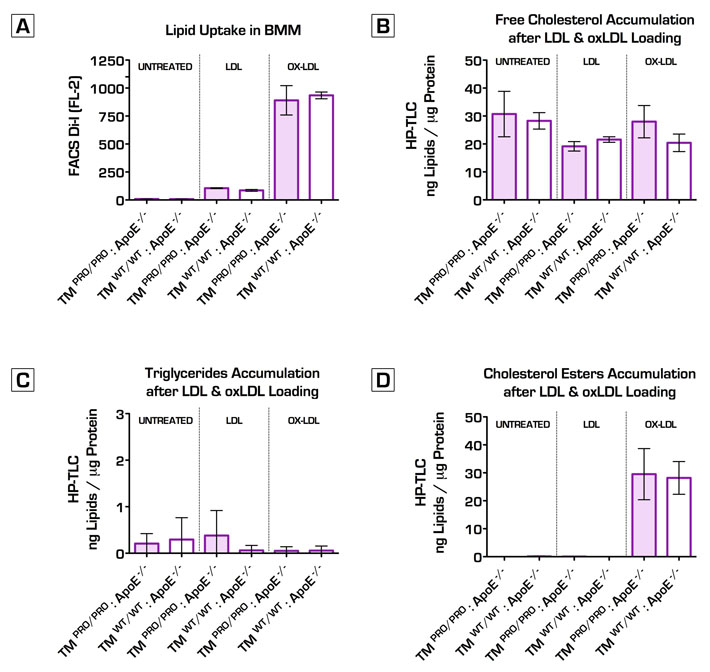

Supplement: Figure S1 — Hypercoagulability in TMPro/Pro:ApoE−/− mice does not alter lipid uptake in bone marrow-derived macrophages (BMM). (A) There were no significant differences found in the lipid uptake in BMM derived from TMPro/Pro:ApoE−/− and control ApoE−/− mice, as determined by flow cytometry analysis. (B, C, D) In addition, we also used high performance thin layer chromatography to test the free cholesterol, cholesterol esters and triglycerides accumulation in BMM in response to LDL and oxidized LDL loading and there were no significant differences detected between BMM obtained from TMPro/Pro:ApoE−/− and control ApoE−/− mice. Error bars represent mean ± SD. Abbreviations: HP-TLC - high performance thin layer chromatography; BMM - Bone marrow-derived macrophages; LDL – low-density lipoprotein; oxLDL – oxidized low-density lipoprotein. (DOC) [file pone.0055784.s001.doc]

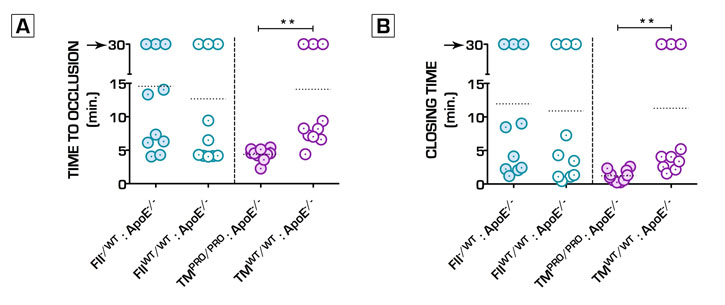

Supplement: Figure S2 — 20% FeCl3-induced arterial injury in hyper- and hypocoagulable atherosclerosis-prone mice. Time to occlusion (TTO) and closing times (CT) were established. TTO is defined as the time after FeCl3 application required for the blood flow to decline to 90%, whereas CT represents the time from the start of flow reduction to a complete occlusion of the carotid artery. (A, B) Both TTO and CT were significantly shortened in TMPro/Pro:ApoE−/− as compared to ApoE−/− control mice (TTO: 4.4±0.9 vs. 14.1±11.1 min., respectively; n = 10 per group, p = 0.0010) (CT: 1.2±0.8 vs. 11.3±13.0 min., respectively; n = 10 per group, p = 0.0010), suggesting for a pro-thrombotic arterial vessel wall phenotype. In contrast, hypocoagulability in FII−/+:ApoE−/− mice had no effect on thrombus formation during FeCl3-induced arterial injury. Of note, all 10 out of 10 of the TMPro/Pro:ApoE−/− mice formed an occlusive thrombus (animals depicted at 30 min. represent all mice, which did not induce occlusive thrombus formation, indicated by an arrow). *p<0.05; **p<0.01; ***p<0.001. Dotted lines represent mean. (DOC) [file pone.0055784.s002.doc]

**
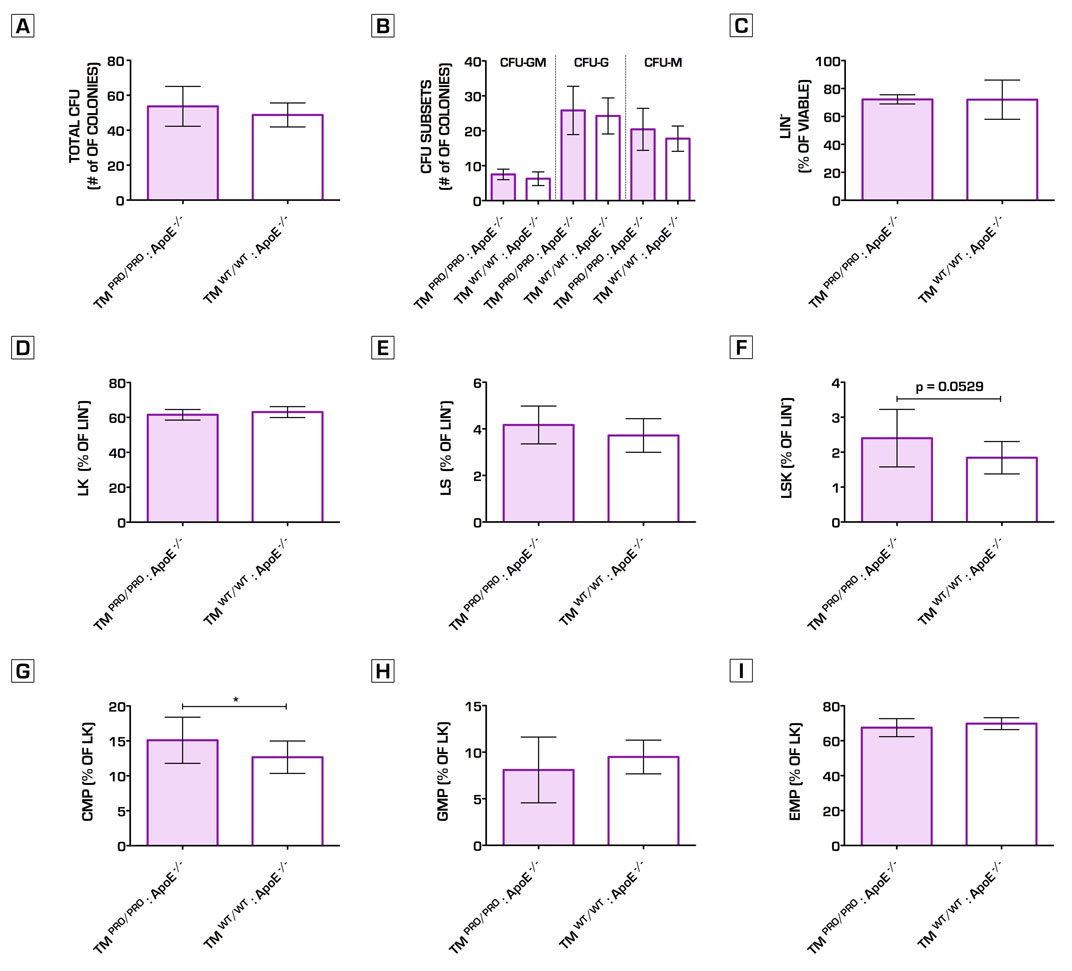
**

**
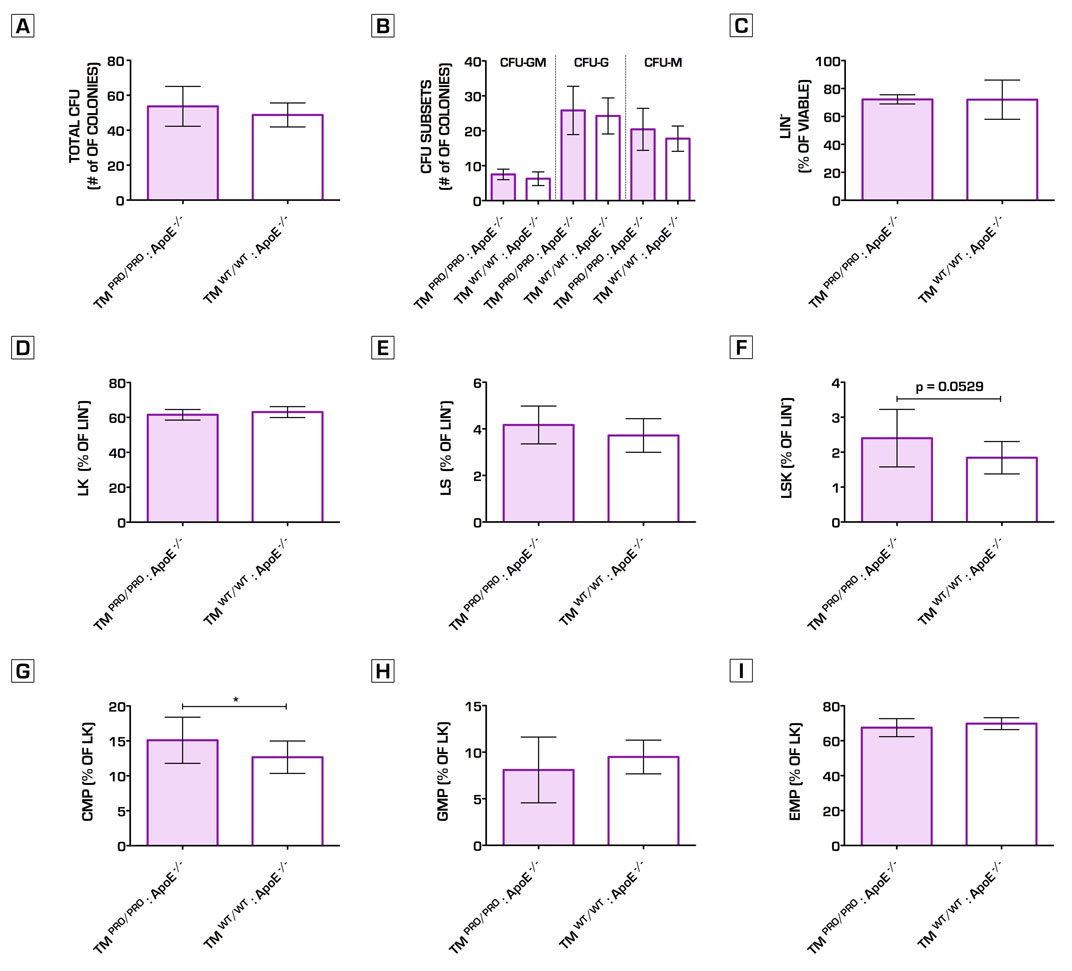
**

Supplement: Figure S3 — The effects of hypercoagulability on hematopoiesis. Using a CFU-C (colony forming unit in culture) assay, we established that there were no significant differences in the amount of total colonies produced by TMPro/Pro:ApoE−/− as compared to ApoE−/− control mice after 8 weeks on a regular chow diet (A). Furthermore, we could not find any changes in the composition, as determined by the CFU subset analysis, indicating that hypercoagulability does not affect hematopoiesis in the bone marrow compartment (B). FACS analysis of the bone marrow consolidated the results of the CFU-C assay (C, D, E). The amount of LSK (Lin−/Sca-1+/c-Kit+) cells showed a tendency towards an increase in the TMPro/Pro:ApoE−/− compared to ApoE−/− control mice (4.2±0.8% vs. 3.7±0.7%; n = 12 per group, p = 0.0529) (F). The amount of CMP (common myeloid progenitor) cells was significantly increased in the TMPro/Pro:ApoE−/− mice compared to the controls (15.1±3.3% vs. 12.7±2.3%; n = 12 per group, p = 0.0402). (G). In addition, EMP and GMP populations in the bone marrow remained unaffected by the hypercoagulable state in TMPro/Pro:ApoE−/− mice (H, I). *p<0.05; **p<0.01; ***p<0.001. Error bars represent mean ± SD. Abbreviations: CFU - colony forming unit; GM - granulocyte-macrophage progenitor; G - granulocyte progenitor; M - macrophage progenitor; LK - cells positive for LIN−c-Kit+Sca-1− lineage markers; LSK - cells positive for LIN−c-Kit+Sca-1+ lineage markers; CMPs - common myeloid progenitors; GMP - granulocyte/macrophage progenitors; EMP – erythroid/megakaryocyte progenitors. (DOC) [file pone.0055784.s003.doc]

**
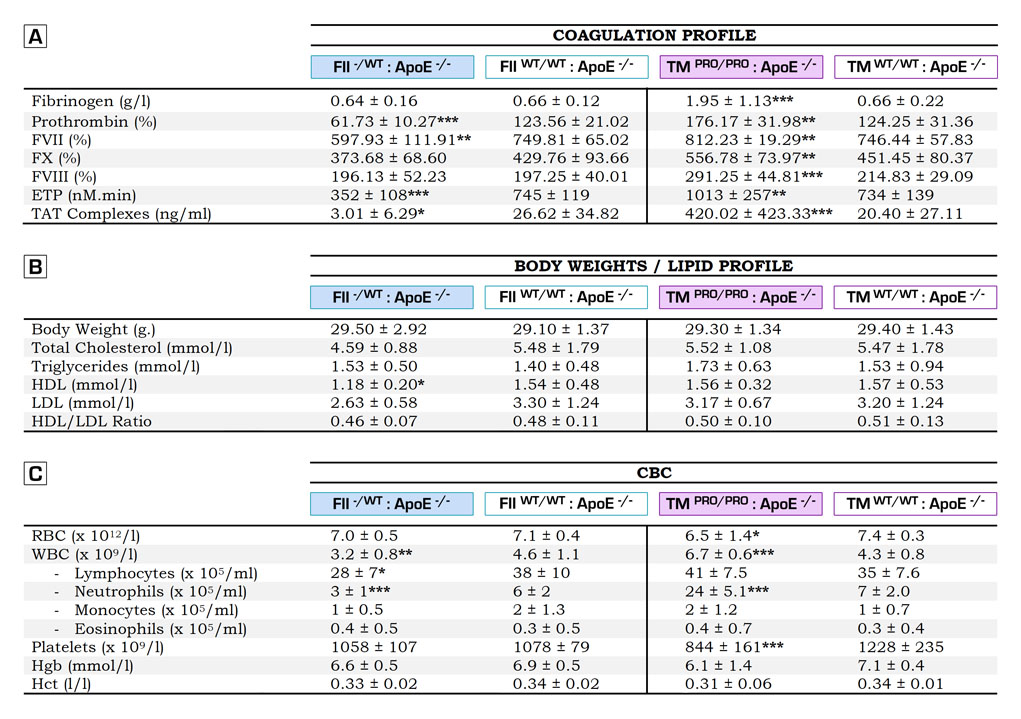
**

Supplement: Table S1 — Coagulation profile (A), body weight, lipid profile (B) and complete blood counts (C), assessed after 35 weeks on regular chow diet in FII−/+:ApoE−/−, TMPro/Pro:ApoE−/− and control ApoE−/− mice (n = 10 per group). *p<0.05; **p<0.01; ***p<0.001. Data are presented as mean ± SD. Abbreviations: ETP – Endogenous Thrombin Potential; TAT – Thrombin-Antithrombin Complex; HDL – High-Density Lipoprotein; LDL – Low-Density Lipoprotein; CBC – Complete Blood Count; RBC – Red Blood Cells; WBC – White Blood Cells; Hgb – Hemoglobin; Hct – Hematocrit. (DOC) [file pone.0055784.s004.doc]

**
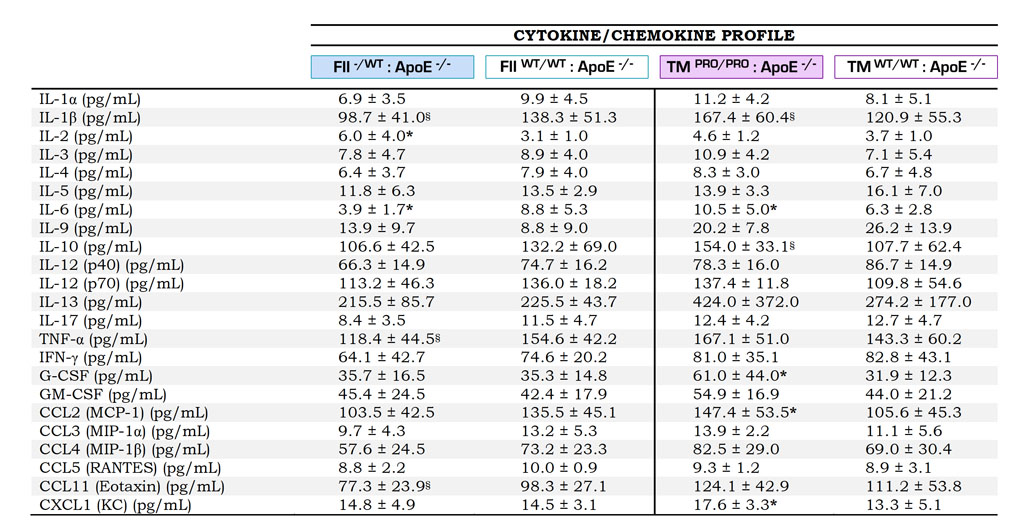
**

Supplement: Table S2 — Cytokine and chemokine profile assessed after 35 weeks on regular chow diet in FII−/+:ApoE−/−, TMPro/Pro:ApoE−/− and control ApoE−/− mice (n = 10 per group). *p<0.05; **p<0.01; ***p<0.001. Data are presented as mean ± SD. Abbreviations: IL – interleukin; TNF-α - tumor necrosis factor-alpha; IFN-γ - Interferon-gamma; G-CSF - Granulocyte colony-stimulating factor; MCP-1 - monocyte chemotactic protein-1; MIP-1α - Macrophage inflammatory protein-1α; MIP-1β - Macrophage inflammatory protein-1β; RANTES - Regulated upon Activation, Normal T-cell Expressed, and Secreted; KC - keratinocyte chemoattractant. (DOC) [file pone.0055784.s005.doc]

**
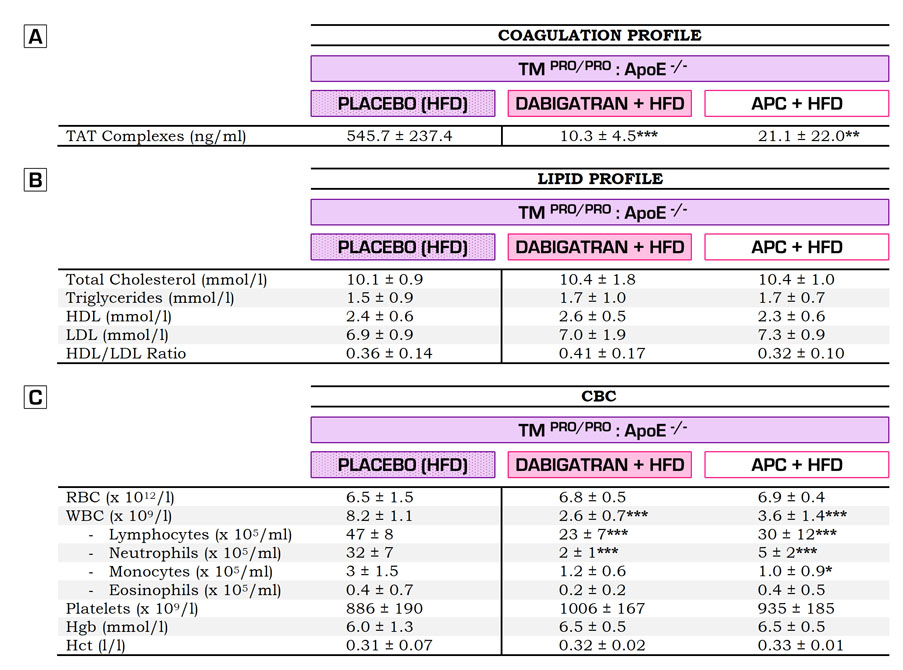
**

Supplement: Table S3 — Coagulation profile (A), lipid profile (B) and complete blood counts (C), assessed at 6 weeks after carotid collar placement in TMPro/Pro:ApoE−/− on high-fat diet and treated with placebo, oral Dabigatran etexilate or mouse recombinant APC (n = 10 per group). *p<0.05; **p<0.01; ***p<0.001 (Intervention groups compared to placebo group). Data are presented as mean ± SD. Abbreviations: TAT – Thrombin-Antithrombin Complex; HDL – High-Density Lipoprotein; LDL – Low-Density Lipoprotein; CBC – Complete Blood Count; RBC – Red Blood Cells; WBC – White Blood Cells; Hgb – Hemoglobin; Hct – Hematocrit; APC – Activated Protein C. (DOC) [file pone.0055784.s006.doc]

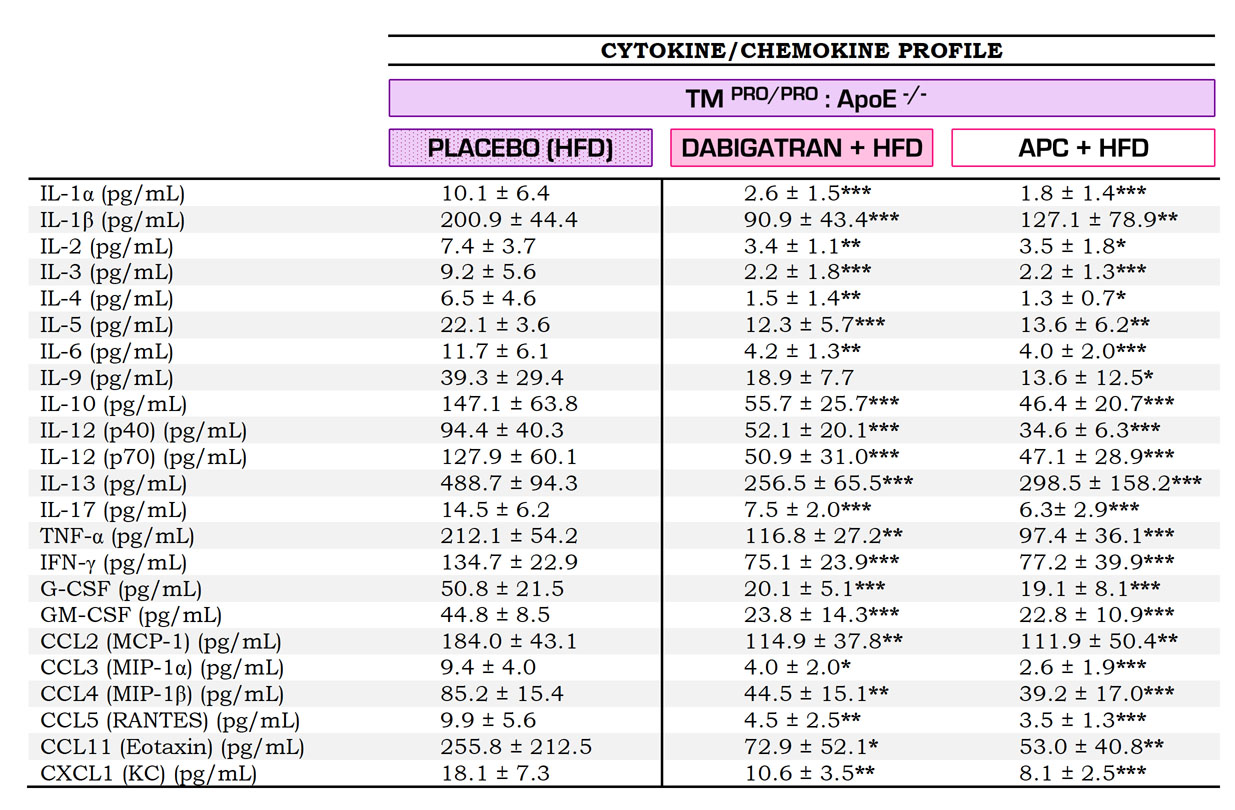

Supplement: Table S4 — Cytokine and chemokine profile assessed at 6 weeks after carotid collar placement in TMPro/Pro:ApoE−/− on high-fat diet and treated with placebo, oral Dabigatran etexilate or mouse recombinant APC (n = 10 per group). *p<0.05; **p<0.01; ***p<0.001 (Intervention groups compared to placebo group). Data are presented as mean ± SD. Abbreviations: IL – interleukin; TNF-α - tumor necrosis factor-alpha; IFN-γ - Interferon-gamma; G-CSF - Granulocyte colony-stimulating factor; MCP-1 - monocyte chemotactic protein-1; MIP-1α - Macrophage inflammatory protein-1α; MIP-1β - Macrophage inflammatory protein-1β; RANTES - Regulated upon Activation, Normal T-cell Expressed, and Secreted; KC - keratinocyte chemoattractant; APC – Activated Protein C. (DOC) [file pone.0055784.s007.doc]
