# Supplementary material for: Genetic and Pharmacological Modifications of Thrombin Formation in Apolipoprotein E-deficient Mice Determine Atherosclerosis Severity and Atherothrombosis Onset in a Neutrophil-Dependent Manner
Source: PLoS One. 2013 Feb 7;8(2):e55784. doi: 10.1371/journal.pone.0055784 (PMC3567111; doi:10.1371/journal.pone.0055784)
Supplement: Methods S1 — (DOC) [file pone.0055784.s008.doc]

**Blood Sampling, Blood Cell Counts, Blood Coagulation and Lipid Profile Analysis.** Peripheral blood samples, obtained via vena cava, were collected in 3.2% sodium citrate (weight/volume). Samples were analyzed on automated hematology analyzer (Beckman Coulter, FL, USA) and the following parameters were established: red blood cell count (RBC), white blood cell count (WBC), platelet count, hemoglobin (Hgb) and hematocrit (Hct). Blood samples were centrifuged for 15 minutes at 4500 rpm at room temperature to pellet blood cells. Plasma was transferred to a fresh tube, centrifuged for additional 5 minutes at 14000 rpm in order to discard remaining cells and debris, and then stored at -80 °C. Differential WBC counts were performed manually on May-Grünwald Giemsa stained blood smears (300 cells/smear) and presented as absolute counts.

Plasma levels of coagulation factors (fibrinogen, prothrombin, FVII, FX, FVIII) were determined via automated Dade Behring Blood Coagulation System (BCS, Siemens Healthcare Diagnostics, Deerfield, IL) using human reagents. Murine thrombin-antithrombin complex (TATc) levels were determined with commercially available micro-enzyme immunoassay kit (Enzygnost® TAT Micro, Siemens Healthcare Diagnostics Products, Marburg, Germany). Using a fluorogenic substrate for thrombin (Z-Gly-Gly-Arg-AMC), plasma thrombin generation was recorded via the Calibrated Automated Thrombogram (CAT) method (Thrombinoscope BV, Maastricht, The Netherlands). Thrombin generation curves and endogenous thrombin potential (ETP) were calculated by the means of Thrombinoscope™ software (Thrombinoscope BV, Maastricht, The Netherlands). Plasma samples were analyzed in duplicates in a 96-well plate Fluoroskan Ascent Reader (Thermo Labsystems, Hensinki, Finland; 390/460 filter set) as previously described. Plasma total cholesterol, triglycerides and HDL cholesterol concentrations were measured in duplicates using automated enzymatic colorimetric assays (CHOD-PAP/GPO-PAP, Roche Diagnostics, Germany). LDL cholesterol concentrations were derived using the Friedewald equation.

**Cytokines and Chemokines Profile Analysis.** Baseline (TM^Pro/Pro^:ApoE^-/-^ and FII^-/WT^:ApoE^-/-^ mice) and post-intervention plasma samples (TM^Pro/Pro^:ApoE^-/-^ - Placebo/DE/APC) were obtained from all experimental groups (n=10 per group). Samples from non-atherosclerotic C57Bl/6 mice (n=10) were collected as an additional reference group to the control ApoE^-/-^ mice (n=20 per group). Cytokines were measured using Bio-Plex Pro™ mouse cytokine 23-plex immunoassay (#M60-009RDPD, Biorad Laboratories, Hercules, CA, USA) in accordance with manufacturer’s manual.

**Tissue Harvesting, Preparation and Morphometry.** In the model studying spontaneous atherosclerosis development, all mice were exsanguinated and then perfused with freshly prepared sodium nitroprusside for 5 minutes (Sigma-Aldrich St. Louis, MO, USA; dissolved in phosphate-buffered saline (PBS), pH 7.4). After an additional perfusion with 1% paraformaldehyde, the aortic tree (including all major branches) was carefully excised and fixed in 1% paraformaldehyde solution overnight. Samples were embedded longitudinally in paraffin blocks and sectioned at 4μm thickness. Histological evaluation of general plaque parameters was performed on 6 consecutive hematoxylin and eosin (HE)-stained sections, with 100μm intervals, thus representing the average distribution of atherosclerotic plaque burden throughout the entire depth of the specimen. An identical approach, using 6 successive cross sections (obtained proximally from the collar, 100μm intervals), was undertaken to describe the atherosclerotic plaque parameters in mice subjected to cuff-induced atherosclerosis. Sections were analyzed on research microscopes (Leica DM3000, Leica Microsystems, Wetzlar, Germany/Zeiss Axioskop/Zeiss Axiovert, Jena, Germany) and captured via a digital camera (Leica DFC 425c/Canon PowerShot G5). Plaque parameters were quantified by two independent investigators using Image-Pro Plus 7.0.1 (Media Cybernetics Inc., Bethesda, MD, USA) and Adobe Photoshop CS5 Extended (Adobe Systems*,* San Jose*,* CA*,* USA) software.

**Histology and Immunohistochemistry.** Longitudinal aortic tree and cross carotid artery sections were immunolabeled with rabbit polyclonal anti-Mac-2 antibody (Galectin-3; ab53082; Abcam, Cambridge, UK) as a specific marker for macrophages, rat monoclonal anti-Ly6G antibody (551459; BD Biosciences, San Jose, CA, USA) to detect granulocytes (mainly neutrophils), rabbit polyclonal to α-smooth muscle actin (ab5694; Abcam, Cambridge, UK) to detect vascular smooth muscle cells, rabbit polyclonal to fibrin(ogen) (ab3426; Abcam, Cambridge, UK). Biotinylated or fluorescently labelled polyclonal anti-rat and anti-rabbit immunoglobulins were used as secondary antibodies (E0468; E0431; 1:200; DakoCytomation Denmark A/S, Glostrup, Denmark; ab6717; Abcam, Cambridge, UK; 553896; BD Biosciences, San Jose, CA, USA; Alexa Fluor 488 and Alexa Fluor 555, Invitrogen). For immunohistochemistry, tertiary antibody streptavidin-Alkaline Phosphatase (AP) (1:200; D0396; DakoCytomation Denmark A/S, Glostrup, Denmark) and/or Vector Red Alkaline Phosphatase Substrate, Vectastain Elite ABC and DAB Peroxidase Substrate Kits were additionally used as appropriate (Vector Laboratories*;* Peterborough*,* UK). Hematoxylin was used as a counterstaining, whereas in the case of immunofluorescence microscopy, slides were mounted using Vectashield with Hoechst-33342 to visualize DNA. In addition, sections were stained with toluidine blue to perform morphometric analysis of the necrotic core area, whereas Perl’s Prussian blue staining was used to detect iron deposition. Sirius red staining determined the relative collagen content in the atherosclerotic lesions.

**FeCl_3_-induced Carotid Artery Injury Model.** TM^Pro/Pro^:ApoE^-/-^, FII^-/WT^:ApoE^-/-^ and control ApoE^-/-^ mice (age, 8-9 weeks; weight, ≈25 gr.; on regular chow diet; n=10 per group) were anesthetized by inhalation of 1.5-2.0% isoflurane and placed in a supine position under a dissecting microscope. Exposure of the left common carotid artery was obtained via a midline cervical incision, and then followed by subsequent blunt dissection. Body temperature was maintained at 37°C using an infrared heating lamp and controlled through a rectal probe. The hemodynamic changes were recorded by using a miniature Doppler flow probe connected to a flow module, equipped with a digital data-acquisition system (IdeeQ, Maastricht, The Netherlands). After extensive rinsing of the surgical site with sterile saline, thrombus formation was induced by the application of a 1x2 mm strip of filter paper, saturated with 20% FeCl_3_ solution, to the adventitial surface of the common carotid arteries. Arterial injury with FeCl_3_ lasted for a total of 3 minutes before filter paper was removed. The carotid blood flow was recorder throughout the entire experiment: at baseline and continuously for 30 min. following FeCl_3_-induced arterial injury.

**Lipid Uptake Analysis in Bone Marrow-derived Macrophages (BMM).** Murine BMM were isolated from femurs of both TM^Pro/Pro^:ApoE^-/-^ and control ApoE^-/-^ mice (n=3 per group), cultured and differentiated into macrophages as described before. BMM from both genotypes were incubated for 24 hours in the presence or absence of 25μg/ml of various LDL types. LDL species were labeled with 1,1′-dioctadecyl-3,3,3′,3′-tetramethylindocarbocyanine perchlorate (DiL) in DMSO and incubated overnight. Lipid uptake was established via flow cytometry using FACSCalibur (BD Biosciences Franklin Lakes, NJ, USA). In a second experimental setup, in order to determine the free cholesterol, cholesterol esters and triglycerides accumulation in BMM in response to LDL and oxidized LDL (oxLDL) loading, high performance thin layer chromatography (HPTLC) was used as demonstrated before.

**Leukocyte-Endothelium Interactions in Atherosclerotic Carotid Arteries.** Female TM^Pro/Pro^:ApoE^-/-^ and control ApoE^-/-^ mice (age, 8-9 weeks; n=6 per group) were fed on high-fat diet for 6 weeks (Hope Farms, Woerden, The Netherlands) *ad libitum*. Mice were anesthetized by an i.p. injection of ketamine/xylazine and the left carotid artery was exposed via a supraclavicular incision and dissection. Non-specific and neutrophil-specific leukocyte labelling was performed by intravenous injection of Rhodamine 6G (R6G; Molecular Probes; Invitrogen, Carlsbad, CA, USA) and monoclonal rat anti-Ly-6G-FITC (551460; BD Biosciences, San Jose, CA, USA). Using intravital epifluorescence microscopy (Zeiss Axiotech), we studied leukocyte/neutrophil rolling and adhesion in carotid arteries in multiple high-power fields (at least 6, 200x magnification). Leukocytes/neutrophils that remained motionless for more than 30 seconds on the carotid artery were defined as adherent.

**Characterization of Bone Marrow Cell Populations by CFU-C Assays and Flow Cytometry.** Nine-week-old TM^Pro/Pro^:ApoE^-/-^ and control ApoE^-/-^ mice (n=12 per group) were used for each of the different experiments. All assays were performed as extensively described before. In short, bone marrow cells were isolated from femurs and tibias. Cells were counted twice using a count chamber and the concentration was calculated for each sample. Per well 10,000 bone marrow cells were added to 1 mL methylcellulose medium with recombinant cytokines (MethoCult GF M3434, StemCell Technologies, Grenoble, France). After incubation for 7 days (37°C; 5% CO_2_) the total number of colonies was quantified by an independent operator, and GM-CFU, G-CFU, and M-CFU colonies were specified based on morphology. In parallel, bone marrow cells were analyzed for progenitor cell composition by flow cytometry. Lineage positive cells were depleted by MACS LS columns and MACS mouse lineage depletion kit (#130-042-401 and #130-090-858, Miltenyi Biotec, Bergisch Gladbach, Germany). The lineage negative population was stained with antibodies against c-Kit (CD117), Sca-1, CD16/32, and CD34 (17-1171, 45-5981, 25-0161, 11-0341; eBioScience, San Diego, CA, USA) to determine relevant subsets and measured on a FACS CantoII flow cytometer (BD BioSciences). Oxidative burst capacity of mature leukocytes in the bone marrow was determined by flow cytometry. Full bone marrow was labeled with antibodies against CD11b (25-0118; eBioScience) and Ly6G (560600; BD BioSciences). Next, cells were incubated with dihydrorhodamine (DHR123; 2 μM; D632; Life Technologies Invitrogen, Bleiswijk, The Netherlands) and subsequently stimulated with phorbol 12-myristate 13-acetate (PMA; 160 nM; P1585; Sigma-Aldrich, St. Louis, MO, USA). Samples were washed and measured within 30 minutes on a FACS CantoII flow cytometer (BD BioSciences).

**REFERENCES**

1. Tchaikovski SN, BJ VANV, Rosing J, Tans G (2007) Development of a calibrated automated thrombography based thrombin generation test in mouse plasma. Journal of thrombosis and haemostasis : JTH 5: 2079-2086.

2. Friedewald WT, Levy RI, Fredrickson DS (1972) Estimation of the concentration of low-density lipoprotein cholesterol in plasma, without use of the preparative ultracentrifuge. Clin Chem 18: 499-502.

3. Groeneweg M, Vergouwe MN, Scheffer PG, Vermue HP, Sollewijn Gelpke MD, et al. (2008) Modification of LDL with oxidized 1-palmitoyl-2-arachidonoyl-sn-glycero-3-phosphorylcholine (oxPAPC) results in a novel form of minimally modified LDL that modulates gene expression in macrophages. Biochimica et biophysica acta 1781: 336-343.

4. Groeneweg M, Kanters E, Vergouwe MN, Duerink H, Kraal G, et al. (2006) Lipopolysaccharide-induced gene expression in murine macrophages is enhanced by prior exposure to oxLDL. Journal of lipid research 47: 2259-2267.

5. Drechsler M, Megens RT, van Zandvoort M, Weber C, Soehnlein O (2010) Hyperlipidemia-triggered neutrophilia promotes early atherosclerosis. Circulation 122: 1837-1845.

6. Akashi K, Traver D, Miyamoto T, Weissman IL (2000) A clonogenic common myeloid progenitor that gives rise to all myeloid lineages. Nature 404: 193-197.
